# Alkaline shear-thinning micro-nanocomposite hydrogels initiate endogenous TGFβ signaling for in situ bone regeneration

**DOI:** 10.1038/s41536-023-00333-z

**Published:** 2023-10-13

**Authors:** Yuting Niu, Zhen Yang, Yang Yang, Xu Wang, Ping Zhang, Longwei Lv, Sainan Wang, Yan Liu, Yunsong Liu, Yongsheng Zhou

**Affiliations:** 1 Central Laboratory, Peking University School and Hospital of Stomatology, Beijing, 100081 PR China; 2 National Center for Stomatology & National Clinical Research Center for Oral Diseases & National Engineering Research Center of Oral Biomaterials and Digital Medical Devices & Translational Research Center for Orocraniofacial Stem Cells and Systemic Health, No. 22, Zhongguancun South Avenue, Haidian District, Beijing 100081 PR China; 3 Department of Prosthodontics, Peking University School and Hospital of Stomatology, Beijing, 100081 PR China; 4 Department of Cariology and Endodontology, Peking University School and Hospital of Stomatology, Beijing, 100081 PR China; 5 Laboratory of Biomimetic Nanomaterials, Department of Orthodontics, Peking University School and Hospital of Stomatology, Beijing, 100081 PR China

**Keywords:** Regenerative medicine, Tissue engineering, Biomedical materials, Chemotaxis

## Abstract

Recruiting endogenous stem cells to bone defects without stem cell transplantation and exogenous factor delivery represents a promising strategy for bone regeneration. Herein, we develop an alkaline shear-thinning micro-nanocomposite hydrogel (10-MmN), aiming to alkaline-activate endogenous TGFβ1 and achieve in situ bone regeneration. It contains polyethyleneimine (PEI)-modified gelatin, laponite nanoplatelets (LAP), a bicarbonate buffer with a pH of 10, and gelatin microspheres (MSs). PEI-modified gelatin plays a pivotal role in hydrogel fabrication. It endows the system with sufficient positive charges, and forms a shear-thinning nanocomposite matrix in the pH 10 buffer (10-mN) with negatively charged LAP via electrostatic gelation. For biological functions, the pH 10 buffer dominates alkaline activation of endogenous serum TGFβ1 to recruit rat bone marrow stem cells through the Smad pathway, followed by improved osteogenic differentiation. In addition, MSs are incorporated into 10-mN to form 10-MmN, and function as substrates to provide good attachment sites for the recruited stem cells and facilitate further their osteogenic differentiation. In a rat critical-sized calvarial defect model, 10-MmN exhibits excellent biocompatibility, biodegradability, hydrogel infusion and retention in bone defects with flexible shapes and active bleeding. Importantly, it repairs ~95% of the defect areas in 3 months by recruiting TGFβR2^+^ and CD90^+^CD146^+^ stem cells, and promoting cell proliferation, osteogenic differentiation and bone formation. The present study provides a biomaterial-based strategy to regulate alkalinity in bone defects for the initiation of endogenous TGFβ signaling, which can be extended to treat other diseases.

## Introduction

Millions of patients suffer from bone defects caused by trauma, diseases, or congenital deformities^1–3^, and the reconstruction of bone defects remains a major challenge for orthopedic and plastic surgeons. Langer and Vacanti in 1993 introduced the modern concept of tissue engineering, which contains three elements—seed cells, growth factors (GFs), and engineered biomaterials^4^. Bone tissue engineering (BTE) can overcome the limitations of autografts and allografts, such as a second invasive surgery with pain, infections, and graft rejection^3,5^. Many studies have combined engineered biomaterials, bone marrow mesenchymal stem cells (BMSCs), and exogenous GFs to repair bone defects^5,6^. However, the manipulation of BMSCs encounters strict technical and regulatory requirements, and the infused ex vivo BMSCs have been reported absent in bone marrow regions^7^. Moreover, the use of exogenous GFs for BMSC-mediated bone regeneration may be accompanied by safety, efficacy, and cost concerns^8^.

It is an ideal strategy to only use engineered biomaterials to manipulate endogenous GFs, stimulating the regenerative potential of native mesenchymal stem cells (MSCs) for in situ bone regeneration^9^. Transforming growth factor β 1 (TGFβ1) ubiquitously exists in cells of multiple types, extracellular matrices, and blood serum^10^. It triggers the Smad signaling pathway for cell homing and proliferation^11^, and promotes bone formation^12^ and wound healing^13,14^. However, activation is required for a newly secreted latent TGFβ1 (LTGFβ1). Previously, we confirmed that a transient and strongly alkaline treatment (pH 10) is an appealing and feasible method to activate endogenous TGFβ1 derived from dentin. Moreover, we developed an injectable alkaline hydrogel to achieve in situ dental-pulp regeneration^15^. Unfortunately, since bone defects usually have flexible shapes and active bleeding, the hydrogel possessing good fluidity and relatively long gelation time (~5 min) cannot be retained at bone defects for in situ bone regeneration.

Injectable shear-thinning biomaterials (iSTBs) are systems that possess rheological properties and can completely fill a space. They can dynamically form a gel and break in aqueous media, having the potential to address these problems. Laponite^®^ XLG is a kind of synthetic silicate nanoplatelets^16^ and they have a disk shape (25 nm in diameter, 0.92 nm in thickness), with negative charges on their faces and pH-dependent positive charges on their edges (positive at pH <9)^17^. Driven by electrostatic forces, laponite nanoplatelets (LAP) can form iSTBs either by self-gelating in deionized (DI) water, constructing the “house-of-card” structure, or physically crosslinking with Type A gelatin having pH-dependent positive charges at pH <7–9^18,19^. However, these existing iSTBs have the fatal drawback of not electrostatically gelating when the pH values of the environment are larger than 9, where both LAP and original Type A gelatin will have negative charges. In that case, they have not been applied for in situ bone regeneration.

**Fig. 1:**
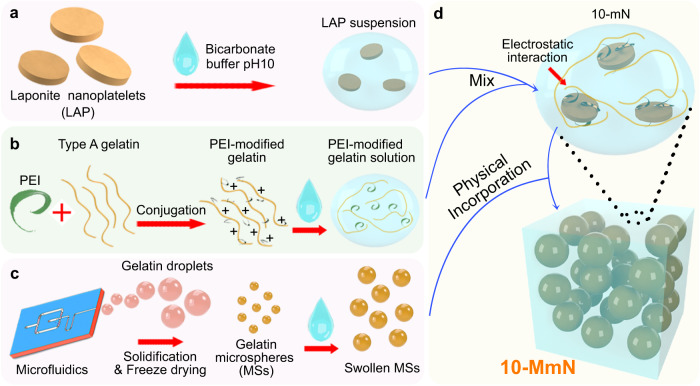
Schematic showing the synthesis of 10-MmN. **a** Laponite nanoplatelets (LAP) were suspended in pH 10 bicarbonate buffer. **b** Polyethyleneimine (PEI) polymers were chemically conjugated onto Type A gelatin backbones to synthesize PEI-modified gelatin, followed by the dissolution in pH 10 bicarbonate buffer. **c** Gelatin droplets were first generated using a microfluidic device. After solidification and freeze-drying, gelatin microspheres (MSs) were swollen in pH 10 bicarbonate buffer. **d** Finally, 10-MmN was yielded by first mixing LAP suspension and PEI-modified gelatin solution to form an alkaline shear-thinning nanocomposite matrix (10-mN), driven by electrostatic interaction, followed by physical incorporation of the swollen MSs.

Herein, we report on an alkaline shear-thinning micro-nanocomposite hydrogel (10-MmN, Fig. 1) specific for in situ bone regeneration. The alkaline hydrogel contains polyethyleneimine (PEI)-modified gelatin, LAP, a bicarbonate buffer with a pH of 10, and gelatin microspheres (MSs). PEI-modified gelatin plays a pivotal role in physical crosslinking of the hydrogel. The attached PEI polymers to the original gelatin molecules provide sufficient positive charges. Therefore, PEI-modified gelatin is able to electrostatically crosslink with negatively charged LAP in the pH 10 buffer, forming a pH 10 shear-thinning nanocomposite matrix (10-mN). 10-mN exhibits injectivity, water resistance, strong cohesion, and hydrogel recovery of the modulus. In addition, MSs, fabricated using a microfluidic method, are incorporated into 10-mN to form 10-MmN, and function as substrates to support cell attachment. Moreover, the pH 10 bicarbonate buffer plays a pivotal role in initiating endogenous TGFβ signaling for bone regeneration. In vitro, the strong alkalinity in 10-MmN activates serum TGFβ1 to induce rat BMSC (rBMSC) migration through the Smad pathway, followed by proliferation and osteogenesis. In vivo, 10-MmN is easily infused and retained in a rat critical-sized calvarial defect. Importantly, it has excellent biocompatibility and biodegradability, and repairs more than 95% of the defect areas in 3 months, by activating endogenous TGFβ1 to recruit TGFβR2^+^ and CD90^+^CD146^+^ stem cells at bone defects, and to promote cell proliferation, osteogenic differentiation and bone formation. Our study provides a biomaterial-based strategy to regulate regional alkalinity at defect areas for the initiation of endogenous cell signaling, without involving transplanted stem cells and exogenous active factors. This strategy can be extended for the treatment of other types of diseases.

## Results

### Synthesis and characterization of 10-mN

**Fig. 2:**
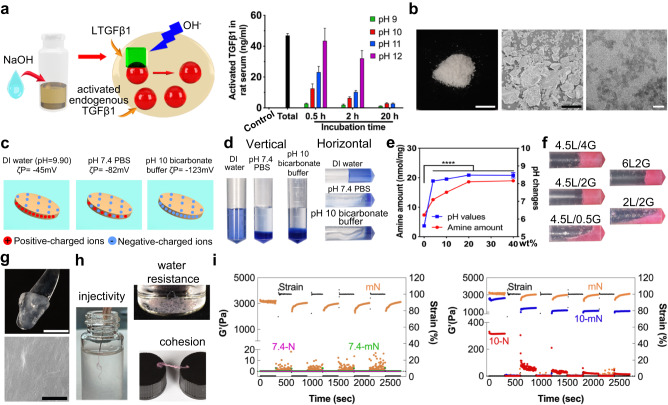
Synthesis and characterization of pH 10 shear-thinning nanocomposite matrix (10-mN). **a** Schematic showing NaOH adjustment for serum TGFβ1 activation and serum TGFβ1 quantification by ELISA. **b** Digital (scale bar: 0.5 cm), SEM (scale bar: 50 μm), and TEM images (scale bar: 50 nm) showing the morphologies of LAP. **c**, **d** Schematic (**c**) and digital (**d**) images showing the gelation properties of LAP in different solvents (stained with blue ink). **e** Quantification of amine groups in PEI-modified gelatin with varied PEI-to-gelatin weight ratios and their alkalinity changes. **f** Digital images showing gelation properties of nanocomposite matrices (with red ink) by inversion experiments. e.g., 4.5L2G represents the nanocomposite matrix having 4.5 wt% of LAP and 2 wt% of PEI-modified gelatin in DI water. **g** Digital (top, scale bar: 0.5 cm) and SEM images (bottom, scale bar: 50 μm) showing the morphology of mN (4.5L2G). **h** Digital images showing injectivity, water resistance, and strong cohesion of mN. **i** Hydrogel recovery of the modulus of 7.4-mN, 7.4-N, 10-mN and 10-N, respectively. mN is the control. Data in (**a**) and (**e**) are presented as mean ± standard deviation.

Abundant free LTGFβ1 exists in the blood, and latent TGFβ1 can be activated by strong acid or strong alkaline, which probably denatures the latency-associated propeptide, thereby disturbing its interaction with TGFβ1 protein dimers^13^. As demonstrated previously^15^, the acidic environment will impact bone repair by destructing hydroxyapatite in hard tissues and activating osteoclasts, and thus only alkaline conditions were investigated in this study. By alkalifying rat serum (Fig. 2a), from pH 9 to pH 12, the amount of activated serum TGFβ1 dramatically increased from 0.16 (control) to 2.8, 12.6, 23.2, and 43.5 ng/ml at 0.5 h intervals, respectively. However, the activated TGFβ1 proteins were slowly degraded with time. In order to balance the activation performance of strongly alkaline materials on TGFβ1 and the damage to surrounding tissues, the pH 10 bicarbonate buffer for 0.5 h treatment was selected in subsequent in vitro experiments. Bicarbonate is one of the most important inorganic salts to function as a buffer system in body fluids, and it has the buffer ability between pH 9 to pH 11. The neutral condition of pH 7.4 phosphate buffer saline (PBS) was used as a control.

Either LAP hydrogels or the nanocomposite matrices containing LAP and original Type A gelatin (OG, the product of irreversible acidic denaturation from collagen) have shown shear-thinning properties. LAP (Na^+^_0.7_[(Mg_5.5_Li_0.3_)Si_8_O_20_(OH)_4_]^−^_0.7_) has a white powder-like gross appearance and a disk-like nano-morphology (Fig. 2b) with little cytotoxicity (Supplementary Fig. 1). Its degradation products (e.g., Mg^2+^, orthosilicates, and Li^+^) are beneficial for bone repair^20,21^, and can be easily taken up by the body^22^ emerging for a variety of biomedical applications^16^. In DI water (Fig. 2c), LAP would have positive charges at the edge by chelating hydrogen ions (H^+^, designated as protonation) from water, and negative charges on the faces, exhibiting a net −45 mV zeta potential (ζP) on the nanoplatelets and a pH of 9.9 in the environment. By electrostatic forces, LAP itself in DI water can spontaneously construct a shear-thinning hydrogel (Fig. 2d). Native tissue matrix has been utilized as scaffolds for tissue regeneration, for example, decellularized matrices are seeded with cells for organogenesis including the heart, lungs, kidney, and liver^23–26^. The primary structure of decellularized tissues is collagen, leading to the idea that collagen or its derivatives, such as Type A gelatin, may serve as scaffolds for tissue defect repair. Since the isoelectric point of OG is pH 7–9, OG will have net positive charges in DI water and gelate with negatively charged LAP, forming iSTBs for various biomedical applications^18,19,27^.

Inorganic ions in buffer solutions can significantly interfere with the charges on either LAP or OG. In pH 7.4 PBS, the positive charges on LAP edges were attenuated, with a reduced net ζP of −82 mV (Fig. 2c). In pH 10 bicarbonate buffers (Fig. 2c), the positive charges were further depleted by alkaline ions, resulting in the net ζP of −123 mV and the failure of shear-thinning hydrogel formation (Fig. 2d). Moreover, in pH 10 bicarbonate buffers, OG will also have negative charges, and OG and LAP will have an inability to electrostatically gelate for iSTBs.

To overcome this problem, we chemically conjugated PEI molecules (MW: 1800 g/mol) onto gelatin backbones to provide sufficient positive charges. PEI polymers have a large number of primary amine groups and exhibit strong positive charges by protonation in a wide range of pHs. Results showed that OG possessed 7.38 nmol/mg amine groups. By adjusting the feed ratios of PEI-to gelatin (4, 10, 20, and 40 wt%), the number of primary amine groups on PEI-modified gelatins significantly increased to 12.56, 15.06, 18.62, and 18.97 nmol/mg (Fig. 2e), respectively. OG showed a pH of 5.61 in a 2% aqueous solution by releasing H^+^. In contrast, both the 20 wt% or the 40 wt% PEI-modified gelatin showed the highest pH value of ~8.48 (Fig. 2e) by intensive protonation, implying excessive positive charges. Compared with the other experiment groups, the 20 wt% PEI-modified gelatin showed strong alkalinity and little toxicity to rat BMSCs (rBMSCs) in 3 days (Supplementary Fig. 2), especially in the highest concentration (100 μg/ml). Therefore, nanocomposite matrices containing LAP and the 20 wt% PEI-modified gelatin sample were used in the following experiments.

We then investigated the rheological properties of nanocomposite matrices. Inversion experiments (Fig. 2f) were conducted to evaluate the gelation features of nanocomposite matrices with varied LAP to PEI-modified gelatin ratios. The critical concentration to form an iSTB was determined to be 4.5 wt% of LAP with 2 wt% of PEI-modified gelatin (4.5L2G) in DI water, designated as “mN”. The control group containing 2 wt% of OG was termed “N”. The mN can be easily picked up using a handpiece, and possessed a uniform nano-morphology (Fig. 2g). It exhibited excellent injectivity, water resistance and strong cohesion (Fig. 2h). We then fabricated the alkaline (pH 10) mN or N by replacing DI water with 0.15 M pH 10 Na_2_CO_3_-NaHCO_3_ buffer. The neutral (pH 7.4) mN or N contained 0.1 M pH 7.4 PBS. As previously demonstrated, the osmolality of the pH 10 bicarbonate buffer is 281 mmol/L, which is within the normal osmolality range (280–310 mmol/L)^15^, benign to normal cells.

To further characterize the shear-thinning properties of different nanocomposite matrices, we tested hydrogel recovery of the modulus after four cycles of high (100%) to low (1%) oscillatory strain amplitudes (Fig. 2i), and mN was used as a standard. mN exhibited the highest initial modulus of ~3000 Pa. At 100% oscillatory strain, the apparent moduli in all groups were weaker than 100 Pa before the end of 5 min of oscillation, demonstrating rapid structure destruction. After the high and low oscillatory strains for four cycles, mN modulus during high strain was 36% weaker than the initial modulus during 100% strain oscillations, and it increased by 31% under quiescent conditions. 10-mN (~2500 pa) showed a stronger initial modulus than 10-N (~320 Pa). The values were 71% and 97% lowered during 100% strain oscillations, and 12% and 89% decreased during low strain, respectively. In contrast, the initial moduli of 7.4-mN and 7.4-N were very weak (lower than 0.3 Pa), and they were further decreased by more than 90% in both high and low-strain conditions. These results indicated that strong negative charges on both OG and LAP in pH 7.4 PBS and pH 10 bicarbonate buffer prevented their gelation. In addition, the complicated electrolytes in PBS could further diminish the stability of nanocomposite matrices. However, PEI modification enabled rapid hydrogel recovery of 10-mN, suggesting appropriate applicability for in situ bone regeneration.

### Synthesis and characterization of 10-MmN

**Fig. 3:**
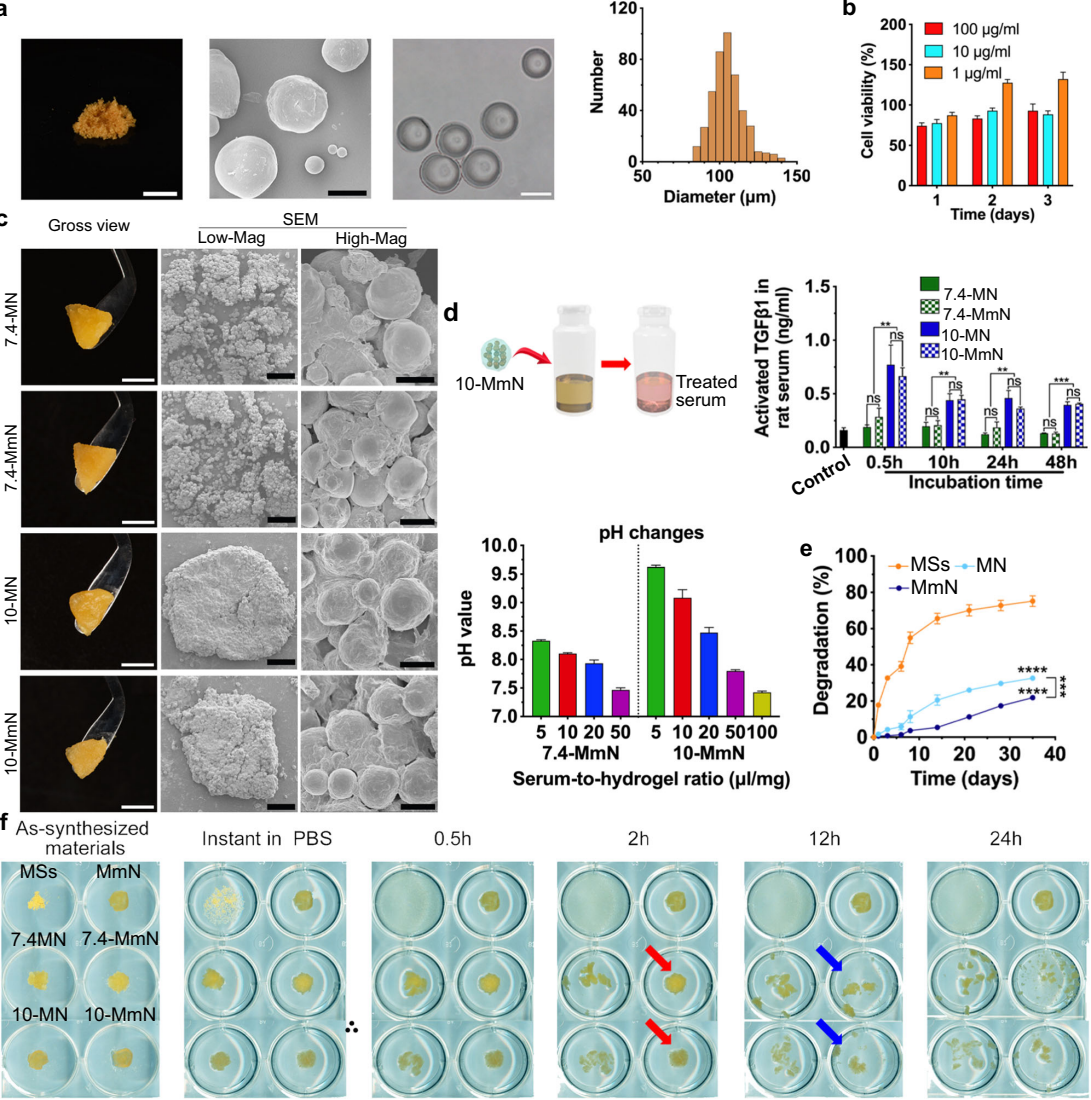
Synthesis and characterization of pH 10 shear-thinning micro-nanocomposite hydrogel (10-MmN). **a** A digital (scale bar: 0.5 cm) and an SEM image (scale bar: 50 μm) showing the morphologies of freeze-dried MSs; a micrograph (scale bar: 100 μm) and the size distribution characterizing swollen MSs. **b** Toxicity of MSs to rBMSCs. **c** Digital (scale bar: 0.5 cm) and SEM images (scale bar: 500 μm for Low-Mag and 50 μm for High-Mag) exhibiting the morphologies of different shear-thinning micro-nanocomposite hydrogels. **d** Serum TGFβ1 activation by hydrogels. Schematic showing 10-MmN treatment to rat serum; activated TGFβ1 using different hydrogels by ELISA; pH changes of 7.4- and 10-MmN in varied serum-to-hydrogel ratios in 2 h, respectively. **e** Enzymatic degradation curves. **f** Dispersion experiments investigating gel integrity and water resistance of different materials by immersing them in PBS. The changes of red arrows to blue arrows indicate the retarded dispersion of both 7.4- and 10-MmN. Data in (**b**), (**d**), and (**e**) are presented as mean ± standard deviation.

After that, the alkaline shear-thinning micro-nanocomposite hydrogel was prepared by incorporating MSs into 10-mN. MSs were synthesized by solidifying gelatin microemulsions, which were generated using a microfluidic device (Fig. 1 and Supplementary Fig. 3). MSs have an orange color and a spherical shape (Fig. 3a). After swelling in an aqueous medium, MSs showed a mean size of ~110 μm. They could act as substrates to support cell growth (Fig. 3b) and adhesion via RGD-containing sequences^15,28,29^. By physical incorporation of swollen MSs into mN, N, 10-mN, 10-N, 7.4-mN and 7.4-N, respectively, MmN, MN, 10-MmN, 10-MN, 7.4-MmN and 7.4-MN were finally acquired (Fig. 3c). All these hydrogels can be picked up with a hand-held instrument, maintaining stable shapes, and they exhibit uniform micro nano-morphologies (Fig. 3c). Notably, these hydrogels can be pre-stored in syringes, convenient for instant use.

Next, the effects of 10-MmN on serum TGFβ1 activation were investigated. We incubated different hydrogels with rat serum (0.16 ng/ml) for varied durations (Fig. 3d). Different from direct adjustment to pH 10 using strongly alkaline solutions, 100 μl of rat serum was alkalified to pH 9.75–9.90 by 20 mg of 10-MmN (Supplementary Table 1). At the 0.5 h interval, ~0.7 ng/ml serum LTGFβ1 was activated by either 10-MmN or 10-MN, about 3 times stronger than 7.4-MmN or 7.4-MN (~0.23 ng/ml). In addition, extended incubation also resulted in TGFβ1 protein degradation to some extent. Therefore, in the following in vitro investigations, we prepared alkaline-treated rat serum by incubating fresh rat serum with different hydrogels for 0.5 h.

It is noted that the increase in the serum-to-hydrogel ratio will cause hydrogel neutralization. We find that 1000 μl of rat serum was able to neutralize 20 mg of the 10-MmN to ~pH 7.8 in 2 h (serum-to-hydrogel ratio of 50 μl/mg, Fig. 3d). Moreover, the 10-MmN was further neutralized to ~pH 7.4 in 2000 μl of rat serum. The pH values in rat serum did not change appreciably during the incubation period from 5 min up to 24 h (Supplementary Fig. 4). Since an adult SD rat possessed 15–35 ml of circulating blood volume or 7–17 ml of serum, the alkaline environment was believed to be gradually and effectively neutralized in a short period of time, avoiding toxic effects.

The stability of shear-thinning micro-nanocomposite hydrogels was then tested. Appropriate biodegradability of engineered biomaterials in wound sites is important for the spatio-temporal growth of neo-tissues. Bare MSs exhibited the quickest digestion in response to gelatinase in PBS, reaching 75% degradation rate in 35 days, because the surface of each MS was in sufficient contact with gelatinase. In contrast, MN degraded by only 33% at the endpoint, and MmN showed significantly retarded degradation by 22%. This was attributed to the much stronger cohesion of mN to wrap MSs, compared to that of N, slowing down the penetration of enzymes (Fig. 3e). In addition, moderate gel integrity and water resistance are beneficial in avoiding the displacement of filled hydrogels upon wound closure. PBS is used to simulate the ionic environment in body fluids to test gel integrity and water resistance (Fig. 3f). Results showed that freeze-dried MSs were immediately swollen and dispersed in PBS, and MmN preserved the integrity until the endpoint. Compared with 10- and 7.4-MNs, 10- and 7.4-MmNs effectively resisted water penetration and maintained gel integrity for more than 2 h, due to the relatively strong cohesion of mN.

### Cytotoxicity, cell migration and osteogenic differentiation behaviors of 10-MmN

**Fig. 4:**
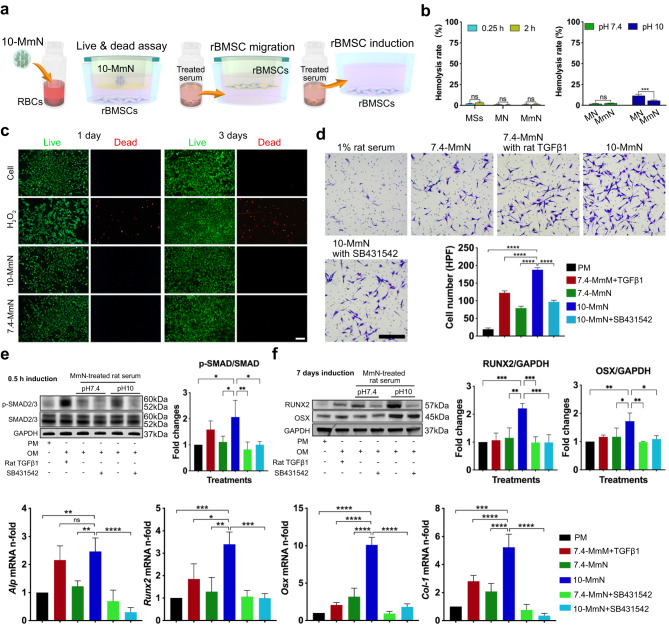
Cytotoxicity, cell migration and osteogenic differentiation behaviors of 10-MmN. **a** Schematic showing hemolysis assay; the co-culture of 10-MmN with rBMSCs to investigate cell viability; the addition of treated serum for rBMSC migration and osteogenic induction. **b** Hemolytic rates. **c** Fluorescence images showing the live (green) and dead (red) rBMSCs. Scale bar: 200 μm. **d** Microphotographs and quantification demonstrating the migration of rBMSCs, induced by culture medium supplemented with 10% 10-MmN-treated rat serum. Scale bar: 50 μm. **e**, **f** The addition of 10% 10-MmN-treated rat serum for (**e**) the smad pathway activation by western blotting and (**f**) osteogenic induction by western blotting and real-time PCR in rBMSCs. Data in (**b**), (**d**), (**e**), and (**f**) are presented as mean ± standard deviation.

To ensure the safety of shear-thinning micro-nanocomposite hydrogels, the cytotoxicity was evaluated. When engineered biomaterials are infused in bone defects with active bleeding, they will directly contact red blood cells (RBCs) and the surrounding bone tissues. In that case, we first investigated the hemolytic rate of Sprague Dawley (SD) rat RBCs (rRBCs) in response to different materials (Fig. 4a). All the materials (Fig. 4b) showed no significant differences in hemolysis at 0.25 h versus 2 h. Although MSs induced a very low hemolytic rate of 2–3.3%, mN lowered the rate to around 1%. Interestingly, MmN balanced the level of hemolysis to 1–1.4%. We then studied the hemolytic effects of 7.4-MmN, 7.4-MN, 10-MmN, and 10-N, respectively. Results showed that pH 7.4 hydrogels were mild to rRBCs with 1.7–2.6% hemolytic rate. However, the pH 10 condition was relatively harmful to rRBCs, and 10-MN resulted in up to 11.5% hemolysis, due to the released alkaline components. Nevertheless, 10-MmN maintained an acceptable hemolytic level of 5.6%, and this would be attributed to its water resistance and strong cohesion, slowing down the diffusion of alkaline substances. The viability of rBMSCs in response to different hydrogels was evaluated by the live & dead assay. First, 7.4-MmN, 7.4-MN, 10-MmN, and 10-MN, respectively, were settled in the upper chamber of transwells, and rBMSCs were seeded in the lower chamber (Fig. 4a). After co-culturing rBMSCs for 1 and 3 days, respectively, negligible dead rBMSCs were found (Fig. 4c and Supplementary Fig. 5). Moreover, the morphologies and viability of rBMSCs remained unaffected after close contact with either MSs, mN or MmN for 3 days, and rBMSCs could attach to the materials (Supplementary Fig. 6). These results indicate good biocompatibility of all the hydrogels at both a pH 10 and a pH 7.4 conditions.

We also evaluated the in vitro biological effects of hydrogel-treated rat serum on rBMSC migration and osteogenic differentiation. Results showed that 10-MmN-treated rat serum resulted in robust rBMSC migration (Fig. 4d). It also strongly activated the Smad signaling pathway by accumulating phospho (p)-Smad2/3 (Fig. 4e, Supplementary Figs. 7–9), and promoted osteogenesis (Fig. 4f, Supplementary Figs. 8 & 9) by upregulating the protein expression of runt-related transcription factor 2 (RUNX2), as well as osterix (OSX), compared to that of 7.4-MmN. Moreover, osteogenesis-related rat genes (Fig. 4f), including alkaline phosphatase (*Alp*), *Col-1*, *Osx* and *Runx2*, respectively, exhibited consistent trends with the proteins. All these effects were strongly diminished by a TGFβ1 inhibitor (SB431542). In addition, the supplemented recombinant rat TGFβ1 (0.1 ng/ml) in the 7.4-MmN-treated rat serum significantly increased the number of migrated rBMSCs and upregulated osteogenesis-related rat protein and gene expression. The above results demonstrated the potential of 10-MmN to effectively initiate endogenous TGFβ signaling for in situ bone regeneration.

### 10-MmN orchestrates in situ bone regeneration in rat calvarial critical-sized defects

**Fig. 5:**
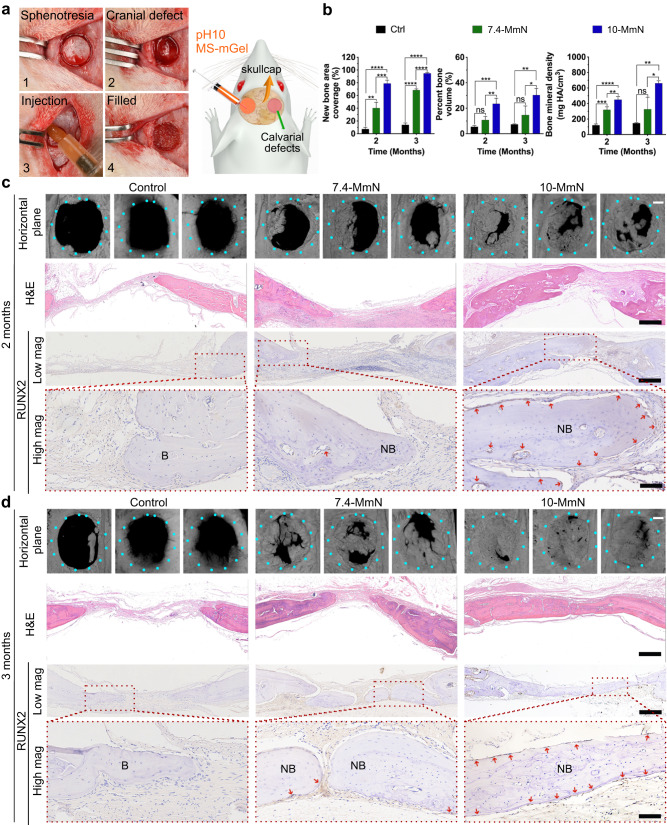
10-MmN achieves nearly complete in situ bone regeneration. **a** Digital images showing the surgery processes (1–4); and schematic indicating the filling of 10-MmN in rat calvarial critical-sized defects with a diameter of 5 mm. **b** μCT analysis of cranial bone morphologies in the calvarial defects after 2 and 3-month treatments, respectively. **c**, **d** Representative μCT-reconstructed 3D (triplicate) images in the horizontal plane (Scale bar: 1 mm); representative H&E staining indicating the histological structures of repaired bone tissues (Scale bar: 500 μm); and immunohistochemical investigations showing RUNX2^+^ osteogenic cells, in the coronal plane after (**c**) 2-month and (**d**) 3-month treatments by 10-MmN, respectively. Scale bar: 500 μm for Low mag and 100 μm for High mag. B bone tissue, NB new bone. Data in (**b**) are presented as mean ± standard deviation.

To confirm the performances of 10-MmN to orchestrate in situ bone regeneration, we carried out animal experiments in a rat calvarial critical-sized defect model. The critical size exceeds the inherent ability of self-regeneration and thus, bone restoration will be postponed and impacted^30^. Sphenotresia was conducted on both sides of the mid-cranial suture in SD rats, and calvarial critical-sized defects of 5 mm diameter were created (Fig. 5a). Then, 10- or 7.4-MmN was injected into the wound sites, and they were shaped with a handpiece (Fig. 5a and Supplementary Fig. 10, Supplementary Video 2). The bone defects without filling were used as a negative control. After 2 months, quantitative data (Fig. 5b) and reconstructed 3D images (Fig. 5c) from micro-computerized tomography (μCT) showed the 10-MmN-infused rat calvarial defects acquired significant bone restoration, with 79% of new bone area coverage (BS/TS), 24% of percent bone volume (BV/TV), and 450 mg HA/cm^3^ bone mineral density (BMD). In contrast, 7.4-MmN led to a small portion of bone repair (BS/TS: 40%, BV/TV: 11%, and BMD: 319 mg HA/cm^3^), and the control group possessed sporadic new bones (BS/TS: 7%, BV/TV: 6% and BMD: 122 mg HA/cm^3^). After 3 months (Fig. 5d and Supplementary Fig. 11), μCT parameters and bone regeneration performance in the 10-MmN group were further enhanced (BS/TS: 95%, BV/TV: 30%, and BMD: 661 mg HA/cm^3^), repairing almost the entire calvarial defects. Although the bone repair was also improved in the 7.4-MmN group (BS/TS: 68%, BV/TV: 15% and BMD: 326 mg HA/cm^3^) and the control group (BS/TS: 14%, BV/TV: 7% and BMD: 148 mg HA/cm^3^), their therapeutic effects were not comparable to 10-MmN.

**Fig. 6:**
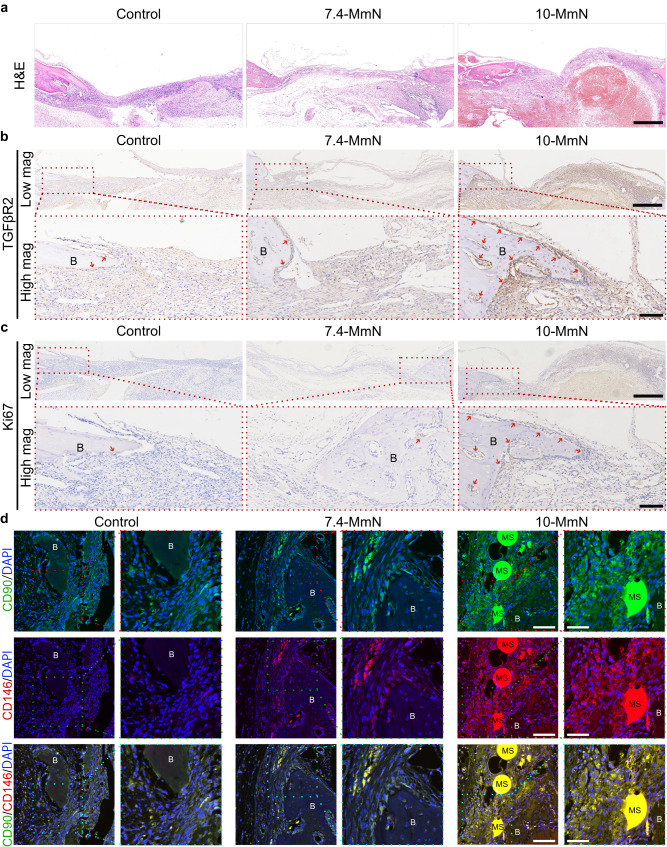
10-MmN repairs bone defects by activating endogenous TGFβ1 to recruit stem cells, followed by accelerated cell proliferation and osteogenic differentiation. After 1-week treatment, decalcified bone tissue sections at rat calvarial defects in the coronal plane were prepared. **a** Representative H&E staining indicating the histological structures of repaired bone tissues by 10-MmN. Scale bar: 500 μm. **b**, **c** Immunohistochemical investigations showing (**b**) TGFβ-receptor 2^+^ (TGFβR2^+^) cells and (**c**) Ki67^+^ proliferating cells. Scale bar: 500 μm for Low mag and 100 μm for High mag. **d** Representative immunofluorescence images showing the positive staining for MSC surface markers of CD90 (green) and CD146 (red). Scale bar: 100 μm for Low mag (left) and 50 μm for High mag (right). Blue DAPI. MS (undegraded) gelatin microspheres. B bone tissue.

Histological investigations depict the story of in situ bone regeneration. After 2 and 3 months of treatments, hematoxylin & eosin (H&E) staining (Fig. 5c and Supplementary Fig. 11) showed accelerated bone restoration in the 10-MmN group. The cells lining new bones were strongly positive for RUNX2 (Fig. 5c, d and Supplementary Fig. 12), demonstrating active bone reconstruction. Cell behaviors in response to short-term treatment (1 week) of 10-MmN were also carefully studied (Fig. 6). At bone margins and bone defect areas (Fig. 6a), the strong cell staining of TGFβ-receptor 2 (TGFβR2, Fig. 6b and Supplementary Fig. 12) showed the initiation of endogenous TGFβ signaling and TGFβ1-responsive cell migration. The Ki67-positive staining (Fig. 6c and Supplementary Fig. 12) indicated the proliferating cells. Moreover, MSC recruitment was identified from the double positive staining of typical MSC surface markers (Fig. 6d) of CD90 and CD146. Besides, some undegraded MSs were observed in the early stage (1 week). On the contrary, 7.4-MmN led to weak staining or few positive cells in both immunohistochemical and double immunofluorescence investigations. The long- and short-term in vivo results illustrated that 10-MmN can initiate endogenous TGFβ signaling to achieve in situ bone regeneration.

## Discussion

**Fig. 7:**
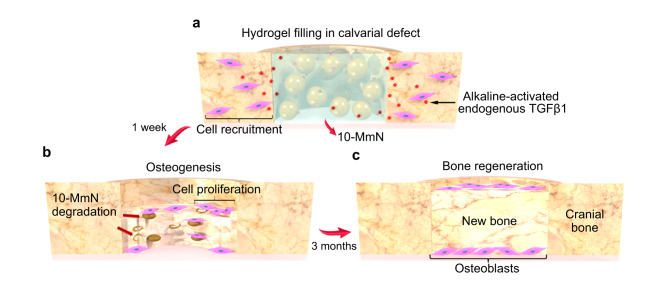
Schematic showing the processes of in situ bone regeneration by 10-MmN. **a** After the injection of 10-MmN in rat calvarial critical-sized defects, free of transplanted seed cells and exogenous growth factors, TGFβ1 can be alkaline-activated for cell recruitment. **b** In a short period of time (one week), 10-MmN will be slowly degraded; the recruited stem cells in bone defects will proliferate, followed by osteogenic differentiation. **c** Three months later, in situ bone regeneration can be achieved, showing neo-bone tissues with bone lining cells on bone surfaces.

Restoration of bone defects with high efficiency remains a major challenge in clinical medicine. BTE is a promising strategy to repair bone defects, and at least two of the three elements of BTE (i.e., engineered biomaterials, seed cells and growth factors) are involved in the published research^5,31,32^. Although current BTE strategies have acquired certain achievements, they are still encountering some problems. Transplanted seed cells would suffer a limited migration capacity and a dramatic reduction in the survival rate within the first few days. This is mainly attributed to a supply shortage of oxygen and/or nutrients, and damage of free radicals to the cells^33,34^. Moreover, the use of exogenous GFs^9,31,35,36^ requires high production costs, and it may cause unexpected side effects, such as carcinogenicity^8^, due to individual differences in sensitivity to GFs. In the present study, we have overcome these problems by avoiding the involvement of either transplanted stem cells or exogenous GFs. 10-MmN possessing strong alkalinity (pH 10) initiated endogenous TGFβ signaling to mobilize the body’s regenerative potential for in situ bone regeneration; and its shear-thinning properties were indispensable for stable filling in bone defects with active bleeding and flexible shapes, for example, superficial defect depth and obscure defect boundary (Fig. 7).

Regional alkaline environments have shown benefits for biological effects. Alkaline intracellular pH activates AMPK–mTORC2 signaling to promote cell survival^37^. Alkaline treatments accelerate platelets to release platelet-derived growth factor (PDGF) and TGFβs^38^. Osteoblasts exhibit increased viability at alkaline pHs^39^. Moreover, we previously have shown the alkaline condition of pH 10 is superior to activating endogenous TGFβ1 to recruit stem cells for dental-pulp regeneration^15^. In contrast, the extracellular acidic conditions can activate osteoclasts, and cause calcium loss and severe disruption of bone microarchitecture^40^. Therefore, in the present study, we engineered the pH 10 bicarbonate buffer in 10-MmN. Alkaline ions could slowly diffuse to the entire wound sites for effective alkaline activation of endogenous TGFβ1, which recognized TGFβ receptors on the stem cells with double positive MSC surface markers of CD90 and CD146. TGFβ signaling transduced through the Smad pathway^41^, showing increased phosphorylation of Smad2/3. TGFβs affected cell migration by recruiting TGFβR2^+^ stem cells, and determined cell proliferation, exhibiting Ki67^+^ cell staining. Moreover, TGFβ signaling has widely recognized roles in bone formation^42^. The upregulation of downstream key transcription factors of RUNX2 and OSX in stem cells indicated osteogenic differentiation^43,44^. In addition, the enhanced gene expression of Col-1 and Alp in BMSCs confirms collagen synthesis and biomineralization, respectively^45^.

Some researchers have initiated iSTBs containing LAP and OG in pure water environments for broad biomedical applications, such as hemostasis, endovascular embolization^18,19^, drug delivery, as well as tissue repair^27^. However, all these existing iSTBs cannot satisfy the requirements of this project, since in strongly alkaline conditions (e.g., pH 10), the negative charges on both LAP and OG will repulse their physical contacts, thus losing shear-thinning properties. With the existence of PEI polymers in the system, the sufficient positive charges on PEI-modified gelatin drove successful gelation with negatively charged LAP in pH 10 buffer, forming a shear-thinning nanocomposite matrix. 10-MmN exhibits excellent injectivity, strong cohesion, and hydrogel recovery of the modulus (Fig. 2), and importantly it resists water dilution for at least 2 h (Fig. 3). Therefore, 10-MmN can be easily injected into bone defects with active bleeding and flexible shapes. Since LAP can induce blood clotting^19^, 10-MmN would be quickly wrapped by the blood clots inside the bone defects, and the fractures of 10-MmN would not penetrate into the blood capillary and not cause side effects by forming intravascular blood clots. In 10-MmN, the molecular weight of PEI polymers determines both the quantity of positive charges on and cytotoxicity of PEI-modified gelatin. PEI polymers are recognized as one type of promising cationic vectors for gene delivery, and they have a wide range of molecular weights^46^. The higher the molecular weight of a PEI polymer, the more positive charges and cytotoxicity it possesses at the same time. The PEI polymer with a molecular weight of 1800 g/mol was used in this study, showing satisfactory therapeutic achievements while having negligible side effects.

Appropriate shear-thinning properties ensure that 10-MmN can be effectively injected and retained in bone defects. However, it is worth mentioning that the overall mechanical strength of the pH 10 shear-thinning nanocomposite matrix is as low as ~3000 Pa. Although 10-MmN has been successfully used to fill bone defects up to 5 mm and achieve bone regeneration, it is not suitable for use in load-bearing sites that may need to withstand pressures to several GPa.

In this study, 10-MmN led to 0.7 ng/ml of active TGFβ1 in rat serum, which was not as strong as directly adjusting the serum to pH 10 (12.6 ng/ml). Interestingly, this has not influenced the therapeutic effects, and both in vitro data of enhanced osteogenesis and in vivo results of in situ bone regeneration confirmed the effectiveness. This is because that low-dose TGFβ1 has been biologically effective for endogenous TGFβ signaling initiation, while high-dose TGFβ1 may impair MSC-mediated bone regeneration while promoting chondrogenesis^47^.

By comprehensive investigations, the alkaline hydrogels exhibited excellent short-term biosafety, including negligible toxic effects of MSs, LAP, and mN on rat BMSCs, respectively, and low hemolytic rates. In addition, the implanted hydrogels were able to degrade in 2 months, and they induced satisfactory bone regeneration in 3 months. These excellent characteristics are derived from the intrinsic properties of LAP and gelatin. Laponite nanoplatelets are non-cytotoxic both in vivo and in vitro^48^, and in 20–50 days, they can naturally dissociate into osteogenesis-related ions (Li^+^, Mg^2+^, and silicates) in physiological environments^49^. It has been extensively reported that gelatin is highly biocompatible and biodegradable without toxicity and immunogenicity^50^. More importantly, the hydrophilic nature of gelatin contents can repel protein (e.g. BSA) and platelet adhesion, avoiding platelet activation and thrombosis^51^. Of course, more biocompatibility evaluations for up to 6 months should be conducted in the future since they are beneficial in further understanding the alkaline hydrogels for clinical application.

Further development of the alkaline activation approach is beneficial for a variety of applications. Optimizing the component and quantity of alkaline substances in hydrogels may lead to further enhanced stem cell homing and in situ bone regeneration. The high-dose-activated endogenous TGFβ1 may be used for cartilage regeneration. In addition, since iSTBs exhibit excellent water resistance, the hydrogels are expected to function in underwater environments, such as arthroscopic operations. Moreover, due to the multiple bio-functions of TGFβ1, the 10-MmN may have extended applicability, for example, skin regeneration.

In summary, we develop an alkaline shear-thinning micro-nanocomposite hydrogel of 10-MmN. It contains PEI-modified gelatin, laponite nanoplatelets, pH 10 bicarbonate buffer, and gelatin microspheres. The 10-MmN exhibits injectivity, water resistance, strong cohesion, and hydrogel recovery of the modulus in strongly alkaline conditions. Moreover, it initiates stem cell recruitment and promotes cell proliferation and osteogenesis by endogenous TGFβ signaling. Using a rat critical-sized calvarial defect model, we further show that this alkaline hydrogel could effectively repair almost the entire defects by activating endogenous TGFβ1 to recruit TGFβR2^+^ and CD90^+^CD146^+^ stem cells at bone defects. Our study provides a biomaterial-based strategy to regulate regional alkalinity to initiate endogenous cell signaling, without involving either transplanted stem cells or exogenous growth factors. This strategy can be extended for the treatment of other types of diseases.

## Methods

### Synthesis of polyetherimide (PEI)-modified gelatin

PEI modification was used to provide abundant amine groups on gelatin backbones. Briefly, 0.9 g of Type A gelatin (90000 g/mol, Sigma) and PEI polymers (1800 g/mol, Aladding) were dissolved in 100 ml of 0.1 M 2-(N-morpholino) ethanesulfonic acid (MES, Aladding) buffer, pH 6 at 37 °C. PEI was added at PEI-to gelatin feed weight ratios of 0, 4% (36 mg), 10% (90 mg), 20% (180 mg) and 40% (360 mg), respectively. The stabilizer of N-hydroxysuccinimide (NHS, Sigma–Aldrich) and the catalyst of N-(3-Dimethyl aminopropyl)-N’-ethylcarbodiimide hydrochloride (EDC, Sigma) were then added at a ratio of 0.213 mg and 0.128 mg per milligram of PEI, respectively. The mixtures were stirred at 37 °C for 16 h, and they were dialyzed against DI water in 12–14 kDa MWCO dialysis tubing (Sigma) for 3 days. Finally, PEI-modified gelatin was freeze-dried for at least 3 days and kept at room temperature.

### Quantification of amine groups and alkalinity

The more primary amine groups attached to gelatin polymers, the stronger the alkalinity of modified materials. The quantity of attached amine groups can be determined by ninhydrin assay. Briefly, 100 μl of 20 mg/ml original Type A gelatin (OG) and different PEI-modified gelatin aqueous solutions, respectively, were mixed with 100 μl of 20 mg/ml ninhydrin solution in dimethyl sulfoxide (DMSO, Aladding), and they reacted at 90 °C to achieve a chromogenic reaction for 20 min. Then, 800 μl of fresh DMSO was mixed to terminate the chromogenic reaction. The optical density (OD) at 570 nm was tested using a microplate reader (ELX808, BioTek, VT). Glycine aqueous solutions from 2.5 to 25 mM were prepared to draw a standard curve. The pH values of 5% OG and different PEI-modified gelatin solutions in DI water were detected by pH meter.

### Synthesis and characterizations of nanocomposite matrices

LAP powder was first dispersed in DI water to acquire a 4.5% LAP suspension. In addition, 0.15 mol/L pH 10 Na_2_CO_3_-NaHCO_3_ buffer and 0.1 mol/L pH 7.4 phosphate buffer saline (PBS) were also used to disperse LAP, maintaining either an alkaline or an acidic environment. In order to evaluate the stability of LAP suspensions, a drop of blue ink was added for staining.

Nanocomposite matrices were fabricated by mixing LAP suspension with either PEI-modified gelatin solution (mN) or original gelatin solution (N) in varied ratios in DI water. In order to evaluate the stability of nanocomposite matrices, a drop of red ink was added for staining.

After sample preparation, inversion experiments were conducted. Briefly, 1 ml of LAP suspensions or nanocomposite matrices were infused in 2 ml microcentrifuge tubes, and they were settled either in a vertical or horizontal position. In addition, nanocomposite matrices were either injected into the water or bridged over two bottle caps. Digital images were taken to record their status. Morphologies of LAP, nanocomposite matrices, and different hydrogels were examined by SEM (HITACHI, SU8010) and TEM (JEOL 1400).

### Design and fabrication of a microfluidic flow-focusing chip

The microfluidic chip was blueprinted using Solidworks CAD software (Dassault Systems). To improve the production efficiency of gelatin droplets, two parallel microfluidic flow-focusing circuits were designed in one chip (Supplementary Fig. 1). The channel width is 150 microns and it is expanded to 500 microns at the outlet region, avoiding the blockage of microemulsions. In each microfluidic circuit, two fluidic ports were opened up using a drill press (Ellis) for tubing connection. A computer numerical control (CNC) milling program was established using Mastercam (CNC Software), and the microfluidic circuits were micromachined on a 3 mm thick PMMA sheet using a CNC machine (HAAS Super Minimill). The microfluidic chip was thoroughly cleaned by sonication and dried using nitrogen purging prior to use. Finally, the circuits were sealed using clear adhesive tape, followed by covering a glass plate.

### Fabrication of gelatin microspheres

Gelatin microemulsions were first fabricated using the microfluidic flow-focusing device. The syringes containing the oil phase (20 wt% Span 80 in mineral oil) and the aqueous phase (5 wt% Type A gelatin solution), respectively, were connected with fluidic ports, using plastic tubing (0.8 mm inner diameter and 1.6 mm outer diameter, C-Flex^®^ laboratory tubing, Sigma). The surrounding temperature was maintained at around 30 °C to prevent the gelatin solution from pre-gelation. The syringes were equipped with syringe pumps (New Era) to control the flow rate of the oil phase at 18.8 μl/min and the aqueous phase at 3 μl/min. The mixture containing gelatin microemulsions and mineral oil was drained out from the chip outlet and was collected in a container. The gelatin microemulsions were maintained at 15 °C and crosslinked to acquire gelatin microspheres (MSs) by adding 10 ml of 5% glutaraldehyde (Sigma) for at least 5 h. MSs were washed with ethanol, cyclohexane, and deionized (DI) water repeatedly, and the excessive aldehyde groups on MSs were blocked in 25 mM glycine solution (Sigma) for 1 h. MSs were lyophilized and kept at room temperature. In order to swell MSs, 10 mg of MSs were immersed in 50 μl of buffers. The mean size of buffer-swollen MSs was observed using an optical microscope and recorded.

### Preparation of shear-thinning micro-nanocomposite hydrogels

Different shear-thinning micro-nanocomposite hydrogels were fabricated by physical incorporation of 10 mg of buffer-swollen MSs and a nanocomposite matrix, which contains 4.5 wt% LAP and 2 wt% PEI-modified gelatin to acquire MmN or original gelatin to acquire MN. For 10-MmN and 10-MN, 0.15 mol/L pH 10 Na_2_CO_3_-NaHCO_3_ buffer was included; and for 7.4-MmN and 7.4-MN, 0.1 mol/L pH 7.4 PBS was used. Their morphologies were tested by SEM and TEM.

### Hydrogel enzymatic degradation

To evaluate the biodegradability, 50 mg of MmN, MN, as well as the equivalent quantity of MSs in them were placed in 2 ml microcentrifuge tubes, respectively. Then, 1 ml of gelatinase (50 μg/ml, collagenase IV, Invitrogen) in PBS was added to digest gelatin components at 37 °C with gentle shaking. At the indicated intervals, each sample was centrifuged at 5000 rpm for 5 min and supernatants (500 μl) were collected. After that, a fresh enzyme solution (500 μl) was refilled to continue the degradation processes. The pure enzyme solution was used as a control. The protein concentration changes were quantified using the BCA method at 562 nm absorbance. Different concentrations of gelatin solutions were used to draw a standard curve.

### Hydrogel recovery of the modulus of nanocomposite matrices

Kinexus Pro^+^ rheometer was used for rheological testing at 37 °C. Typically, 0.26 ml of 10-mN/N and 7.4-mN/N, respectively, were injected into a parallel plate with a 25 mm diameter and 500 μm gap. An appropriate volume of mineral oil was cast around the plate to prevent water evaporation from nanocomposite matrices. Nanocomposite matrices were equilibrated for 10 min before testing, followed by a steady shear at 10 s^−1^ for 2 min to minimize viscosity and eliminate mechanical history. Hydrogel recovery evaluation was conducted at 1 Hz by providing a low strain (1%) for 5 min, followed by providing a high strain (100%) for another 5 min for four cycles to investigate hydrogel recovery. Each analysis was conducted in triplicate.

### Hydrogel dispersion test

The dispersion test was used to investigate the water resistance and cohesion of alkaline shear-thinning micro-nanocomposite hydrogel. Briefly, 20 mg of 10-MN/MmN, 7.4-MN/MmN, as well as the corresponding quantity of MSs and mN, respectively, were settled in 6-well plates, and 2 ml of PBS was added. The time required for the materials to be infiltrated and dispersed at room temperature was monitored.

### Cell culture

Rat bone marrow mesenchymal stem cells (rBMSCs) used in this project were purchased from Cyagen (OriCell^®^ RASMX-01001). Cells between three and five passages were used for further studies. The cells were kept at 37 °C with 95% air, 5% CO_2_, 100% relative humidity. All materials used for cell culture were obtained from GIBCO or Invitrogen, Life Sciences, Life Technologies. The proliferation medium (PM) consists of 10 v/v% fetal bovine serum (FBS), 100 U/ml penicillin G, and 100 mg/ml streptomycin in α-MEM. To prepare an osteogenesis medium (OM), 100 nM dexamethasone, 200 mM L-ascorbic acid, and 10 mM β-glycerophosphate were added to the PM. All in vitro experiments were carried out in triplicate.

### Cell viability

MTT assay was carried out to test the cytotoxicity of materials. One day before the test, 3 × 10^3^ rBMSCs were seeded in each well in 96-well plates. Then, MSs, LAP, original gelatin, and PEI-modified gelatins were added and co-cultured with rBMSCs, respectively. Cell viability was detected by the standard MTT (Methyl thiazolyldiphenyl­tetrazolium bromide, MCE) assay. The absorbance at 570 nm wavelength was tested by a microplate reader, and cytotoxicity was determined by comparing it to cells not exposed to stimulants.

### Live & dead assay

Cytotoxicity of 10-MmN/MN and 7.4-MmN/MN was evaluated by the live and dead assay. First, 1 × 10^5^ rBMSCs were seeded in each well in 24-well plates. Then, 20 mg of 10-MmN/MN and 7.4-MmN/MN were placed in the upper chambers of transwells, respectively, and they were immersed in the culture medium. rBMSCs cultured without any stimulants were used as a negative control, while that with 60 μM H_2_O_2_ was used as the positive control group. After 1 and 3 days, rBMSCs were rinsed with PBS, followed by staining them according to protocols in the Live & Dead Viability/Cytotoxicity Assay Kit for Animal Cells (KGAF001, KeyGen BioTECH). In addition, BMSCs were co-cultured with MSs, mN, and MmN for 3 days, respectively. The morphologies and viability of rBMSCs after close contact with different materials were also investigated. All samples were observed under a fluorescence microscope.

### Hemolysis test

Hemolysis tests were carried out to evaluate the toxicity of 10-MmN/MN and 7.4-MmN/MN to rat red blood cells (rRBCs), respectively. Firstly, the whole blood from 6-to-8-week-old male Sprague Dawley (SD) rats was obtained via cardiac puncture using syringes and pre-conditioned in an EDTA anticoagulant tube. The rRBCs were collected by centrifugation at 2800 rpm for 5 min and washed with PBS to exclude serum and the buffy coat.

To study the influence of time, MmN (20 mg) and the corresponding amount of mN and MSs, together with 100 μl of rRBC suspension were added into 900 μl of PBS, respectively. They were incubated for either 0.25 h or 2 h for comparison, at 37 °C with intermittent shaking. To compare the influences of alkalinity, 100 μl of rRBC suspension and 20 mg of 10-MmN/MN and 7.4-MmN/MN were added into 900 μl of PBS, respectively, incubating 37 °C with intermittent shaking for 2 h. The released hemoglobin in the supernatant was collected, and the optical density (OD) values proportional to hemoglobin concentrations were measured at 540 nm. The rRBCs incubated in PBS and DI water were served as the baseline (0%) and the positive control (100%), respectively. The experiment was repeated three times.

### TGFβ1 activation

Rat serum was first collected. The whole blood was obtained via cardiac puncture using syringes under anesthesia and kept unperturbed at room temperature for at least 30 min. The blood clots were discarded after the centrifugation at 5000 rpm for 10 min at 4 °C. The serum in the supernatant was acquired and kept at −20 °C before use.

To investigate TGFβ1 activation under varied alkaline conditions, appropriate amounts of 1 M sodium hydroxide and 1 M hydrochloric acid were used to adjust the rat serum pH values to be 9, 10, 11, and 12, and they were maintained for 0.5, 2, and 20 h, respectively. The activated TGFβ1 levels were quantified following an ELISA kit (Proteintech).

To evaluate the alkaline-activation performance of different hydrogels, 100 μl of rat serum were incubated with 20 mg of 10- and 7.4-MmN/MN for 0.5, 10, 24, and 48 h, respectively. The serum pH changes were detected using a pH meter (Mettler Toledo).

As a positive control, 25 μl of 1 N HCl was added to 50 μl of rat serum to activate the entire latent TGFβ1, followed by neutralization using 20 μl of 1.2 N NaOH. The untreated rat serum was used as a negative control. Biological triplicates were repeated in all samples. Using a TGFβ1 ELISA assay kit (Proteintech), the OD values at 450 nm and 630 nm were recorded, and the concentrations of activated TGFβ1 were calculated by interpolating into a four-parameter logistic (4PL) standard curve.

In order to monitor the hydrogel neutralization, 20 mg of 10-MmN and 7.4-MmN were immersed in 100, 200, 400, 1000, and/or 2000 μl of rat serum for 5 min, 0.5, 2, and 24 h, respectively. The serum pH changes were detected using a pH meter (Mettler Toledo).

### Cell migration

Cell migration experiments were conducted using a Transwell@ cell culture set with an 8-μm pore size (Corning). Before the tests, rat serum was incubated with 10/7.4-MmN for 0.5 h, respectively, at the ratio of 20 mg of hydrogels to 100 μl of rat serum, followed by neutralization with 1 N HCl. Then, 500 μl of α-MEM supplemented with 10% treated rat serum, respectively, was added to the lower chambers of 24-well plates. Finally, 3 × 10^4^ rBMSCs, after starvation for 12 h, were suspended in α-MEM supplemented with 1% untreated rat serum in the upper chambers of each transwell and incubated for 16 h. In the lower chambers, the α-MEM supplemented with 1% untreated rat serum, 10 % 10-MmN-activated rat serum with 30 μM SB431542 (MCE) or 10% 7.4-MmN -activated rat serum with 0.1 ng/ml recombinant rat TGFβ1 (HY-P70648, MCE) were used as different controls. rBMSCs were then fixed in 4% paraformaldehyde (PFA) in PBS for at least 30 min, and then stained with 0.1% crystal violet for 2 h. rBMSCs migrated to the lower surface of the transwell membrane were imaged and counted under an optical microscope. Biological triplicates were repeated in all samples.

### Cell signaling initiation

To investigate Smad signaling activation and rBMSC differentiation, different culture mediums were prepared, including osteogenesis medium (OM) supplemented with 10% 10/7.4-MmN-treated rat serum, 10% 10/7.4-MmN-treated rat serum with 30 μM SB431542 and 10% 7.4-MmN-activated rat serum with 0.1 ng/ml recombinant rat TGFβ1, respectively. In addition, proliferation medium (PM) supplemented with 10% untreated rat serum was used as a negative control. For Smad signaling activation, rBMSCs were seeded in 6-well plates and incubated for 0.5 h. rBMSCs for osteogenic differentiation were incubated for 7 days.

### Western blotting

For western blotting, cells were first washed with pre-chilled PBS, and RIPA Lysis buffer (Thermo Scientific) was added to harvest proteins in the cell lysates. The buffer contained 1 × phosphatase (Roche Applied Science) and protease (MCE) inhibitor cocktails. Then, cell lysate proteins were separated in a LABLEAD® 4–20% Protein Gel (1.0 mm), transferred to a 0.45 μm polyvinylidene fluoride membrane (Millipore), and blocked in 5 wt% non-fat milk. Primary antibodies, including Phospho-Smad2/3 (8828, CST), Smad2/3 (8685, CST), RUNX2 (ab236639, Abcam), Sp7/Osterix (ab209484, Abcam) and GAPDH (2118, CST) shown in Supplementary Table 2 with a dilution of 1:1000 were covered on the membranes at 4 °C overnight. After incubation with secondary antibodies at room temperature for 1 h, the protein bands were imaged (VILBER, Fusion FX). Then the bands in western blot images were extracted and semi-quantified using the embedded function in the Evolution software, according to the pixels of each band.

### Real-time (RT)-PCR

For quantitative real-time reverse transcription PCR, the addition of Trizol reagent (Ambion®, Life Technologies^TM^, USA) was used to extract the total RNA. Absorbances of 260/280 nm were recorded to determine the quantity and purity of RNAs using a NanoDrop 8000 spectrophotometer. Then, RNA was reverse-transcribed according to the manufacturer’s protocol in the iScript cDNA synthesis kit (Bio-Rad). RT-PCR was performed using SYBR Green Master (Roche, USA) via a Q3 Real-time PCR Detection System (Applied Biosystems). The gene expression of *Col-1, Alp, Runx2, and Osx* were evaluated. The primer sequences are shown in Supplementary Table 3. Relative expression levels were calculated using the 2^−ΔΔCT^ method and determined by normalizing to rat *Gapdh* expression.

### Cell immunofluorescent staining

After seeding 1 × 10^5^ rBMSCs onto 24-mm glass coverslips in 24-well plates, 10% 7.4- and 10-MmN-treated rat serum was supplemented. After 30 min, the attached cells were first fixed with 4% paraformaldehyde (PFA) in PBS for 30 min, blocked in PBS with 5 v/v% rabbit serum and 0.3 v/v% Triton X-100 (93443, Sigma) for 1 h at room temperature, and incubated with Phospho-Smad2/3 antibody (8828, CST) with a dilution of 1:100 in antibody buffer (1 wt% BSA and 0.3 v/v% Triton X-100 in PBS) overnight at 4 °C. Then, the cells were incubated with secondary anti-Rabbit IgG (H + L) F(ab’)2 Fragment (4413, dilution 1:1000, CST) in antibody buffer at room temperature for 1 h. After that, the cells were further incubated with FITC-phalloidin (Sigma–Aldrich) in an antibody buffer at room temperature for another 1 h. Finally, the coverslips were mounted onto glass slides by Fluoroshield™ with DAPI (Sigma). The slides were viewed using a confocal laser scanning microscope (LSM710, Zeiss).

### Animals and animal experiments

The 6-to-8-week-old male SD rats were purchased from the Charles River Company, Beijing, China. All animal experiments were conducted according to the ARRIVE (Animal Research: Reporting of In Vivo Experiments) guidelines. The protocols were approved by the ethics committee of Peking University Health Science Center (Approval number: LA2019019). All the authors comply with all relevant ethical regulations. SD rats were raised under specific pathogen-free (SPF) rooms with a 12-h light/12-h dark cycle. The rats have access to free food pellets and tap water ad libitum.

The sphenotresia was conducted under general anesthesia using 2% pentobarbital sodium (50 mg/kg) in saline by an intraperitoneal route. After the tests for the hind paw withdrawal reflex, the top of the head was shaved with a mini hair trimmer. The appropriate amount of 4% infallible narcosis was regionally injected to enhance local anesthesia, and their eyes were lubricated with viscotears liquid gel. For the surgery, a sagittal skin incision was first made along the midline of the head. The cranial vault was exposed and the periosteal layer overlying the cranial bone was removed using a periosteal separator. A 5-mm defect was drilled on each side of the mid-cranial suture using a trephine burr, under 0.9% saline cooling. After the removal of bone pieces, 10/7.4-MmN, as well as 10/7.4-N were infused into the defects with proper shaping. The defects without hydrogel filling were used as a negative control. Each group has at least 3 animals. By the demix suture method, the soft tissue and skin incision were closed. A recovery cocktail consisting of sterile saline with penicillin (20000 U per rat) and buprenorphine (0.01 mg/ml) was injected intraperitoneally into the rats, which were monitored very carefully in an incubator at 37 °C until recovered. Then, the rats were put back into routine housing. Three rats per group were sacrificed after 1 week, 2 months, and 3 months, respectively, by inhaling excess carbon dioxide, and rat head samples were collected.

### Micro-computerized tomography (μCT) measurement

After sample collection and fixation in 10% formalin for at least 24 h, rat cranial bones were first scanned at a resolution of 12.5 μm using a Skyscan 1174 micro-CT system (Bruker, Belgium). The acquired axial images were integrated in NRecon and CTvox software for visualization and analysis. We defined the entire area of bone defects as the “region of interest” (ROI), and bone volume (BV)/total volume (TV) and bone mineral density (BMD) for each sample were quantified using CTAn software. Bone surface (BS)/tissue surface (TS) was quantified using ImageJ by analyzing μCT-reconstructed 3D images.

### Histological analysis

Rat head samples were collected at required time points, and they were fixed in 10% formalin for at least 24 h. Then they were decalcified in a 10% ethylene diamine tetraacetic acid (EDTA) solution for about 1 month, washed with distilled water, dehydrated, and embedded in paraffin. The tissues were sectioned into slices with 5 μm of thickness and stained by H&E. The immunohistochemical and immunofluorescence studies were conducted by staining the tissue sections using primary antibodies with a dilution of 1:100 and secondary antibodies. The slices were scanned using a tissue section imaging system (Vectra Polaris). The ROIs (region of interest, 1000 × 500 μm, *n* = 3) of each group were randomly designated, and the number of positive cells was recorded. For immunohistochemistry, the primary antibodies include RUNX2 (ab236639, Abcam), TGFβR2 (66636-1-lg, Proteintech), and Ki67 (ab16667, Abcam, Supplementary Table 2). For immunofluorescence, the primary antibodies include CD90 (ab181469, Abcam) and CD146 (ab75769, Abcam, Supplementary Table 2).

### Statistical analysis

Prism 9 (GraphPad) software was used to plot data and analyze statistical differences using unpaired one-way ANOVA, assuming a Gaussian distribution and equal standard deviation (SD). The means of each condition were compared by Tukey’s multiple comparison correction, with a confidence level of 95%. All data are presented as mean ± standard deviation. ^*^P < 0.05, ^**^P < 0.01, ^***^P < 0.001, and ^****^P < 0.0001, *ns* not significant. *n* = 3 independent experiments in all panels.

### Reporting summary

Further information on research design is available in the Nature Research Reporting Summary linked to this article.

### Supplementary information

Supplementary Information

Reporting Summary

Supplementary video 1- Gelatin microemulsion generation

Supplementary video 2- Hydrogel injection and shaping

## Data Availability

All data needed to evaluate or reproduce the conclusions in the paper are present in the paper and/or the Supplementary Materials. Any other requests for raw or processed data will be reviewed by Peking University National Engineering Laboratory for Digital and Material Technology of Stomatology to verify whether the data requested are subject to any intellectual property or confidentiality obligations.
