# Supplementary material for: Alkaline shear-thinning micro-nanocomposite hydrogels initiate endogenous TGFβ signaling for in situ bone regeneration
Source: NPJ Regen Med. 2023 Oct 13;8:56. doi: 10.1038/s41536-023-00333-z (PMC10575889; doi:10.1038/s41536-023-00333-z)
Supplement: Supplementary file 1 — Supplementary Information [file 41536_2023_333_MOESM1_ESM.pdf]

# Supplementary Information

## Supplementary Figures

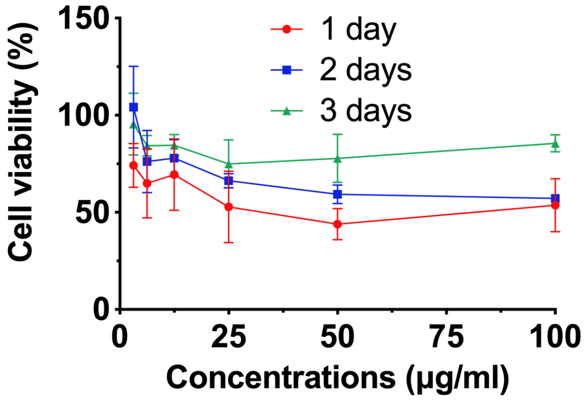


**Supplementary Figure 1.** The toxicity of LAP to rBMSCs. Data are presented as mean+/-standard deviation.

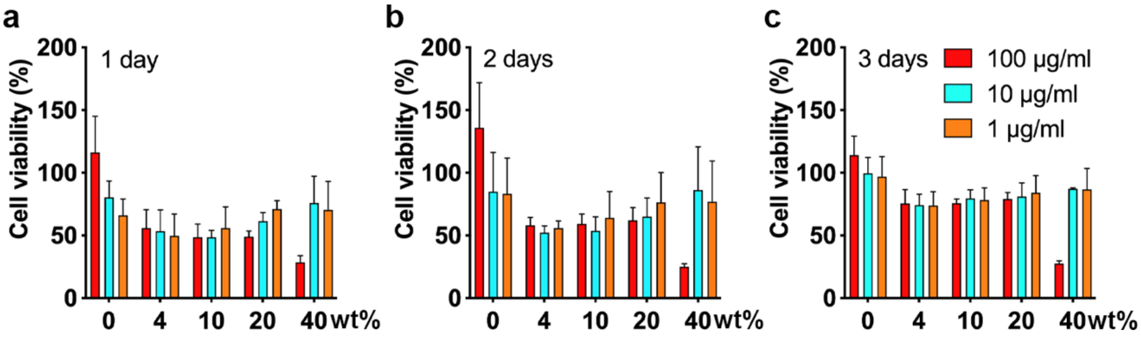


**Supplementary Figure 2.** Toxicity of PEI-modified gelatins to rBMSCs. Data are presented as mean+/- standard deviation.

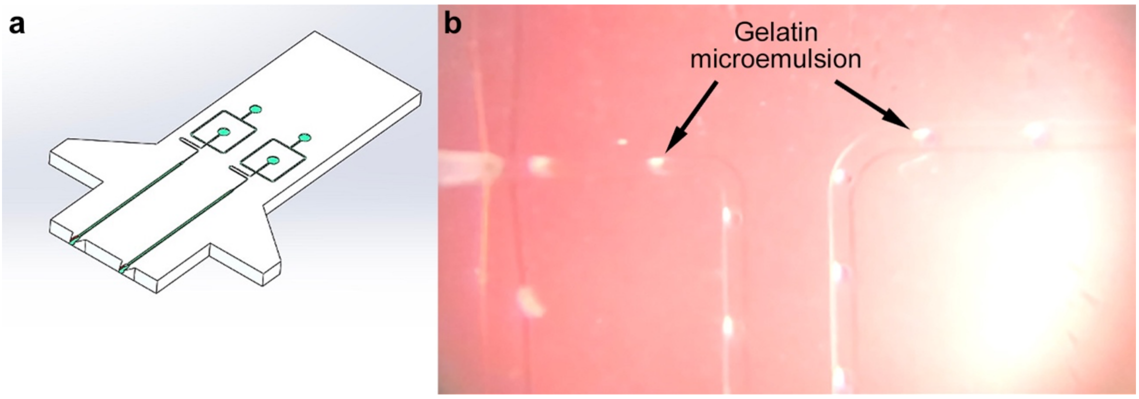


**Supplementary Figure 3.** Schematic showing **a** the microfluidic device with double channels, and **b** the digital image showing generated gelatin microemulsion/droplets in the channels. The movie showing gelatin droplet generation is shown in the Supplementary Video 1.

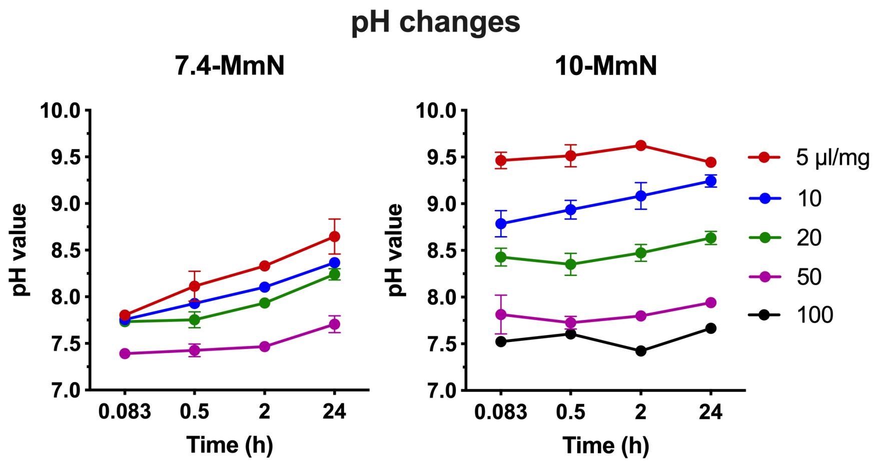


**Supplementary Figure 4.** pH changes of 7.4- and 10-MmN in different serum-to-hydrogel ratios within 24 hours, respectively. Data are presented as mean+/- standard deviation.

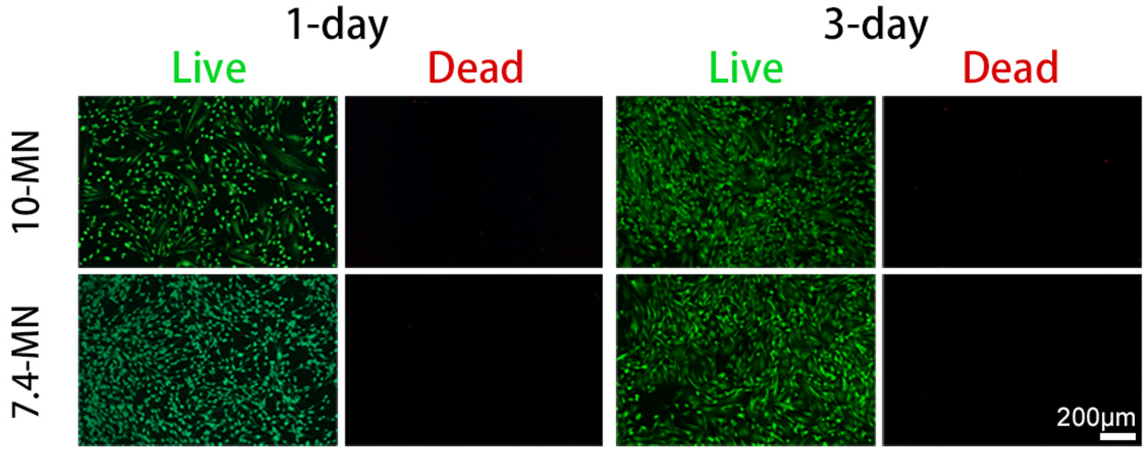


**Supplementary Figure 5.** Fluorescence images demonstrating the live (green) and dead (red) rBMSCs in control groups.

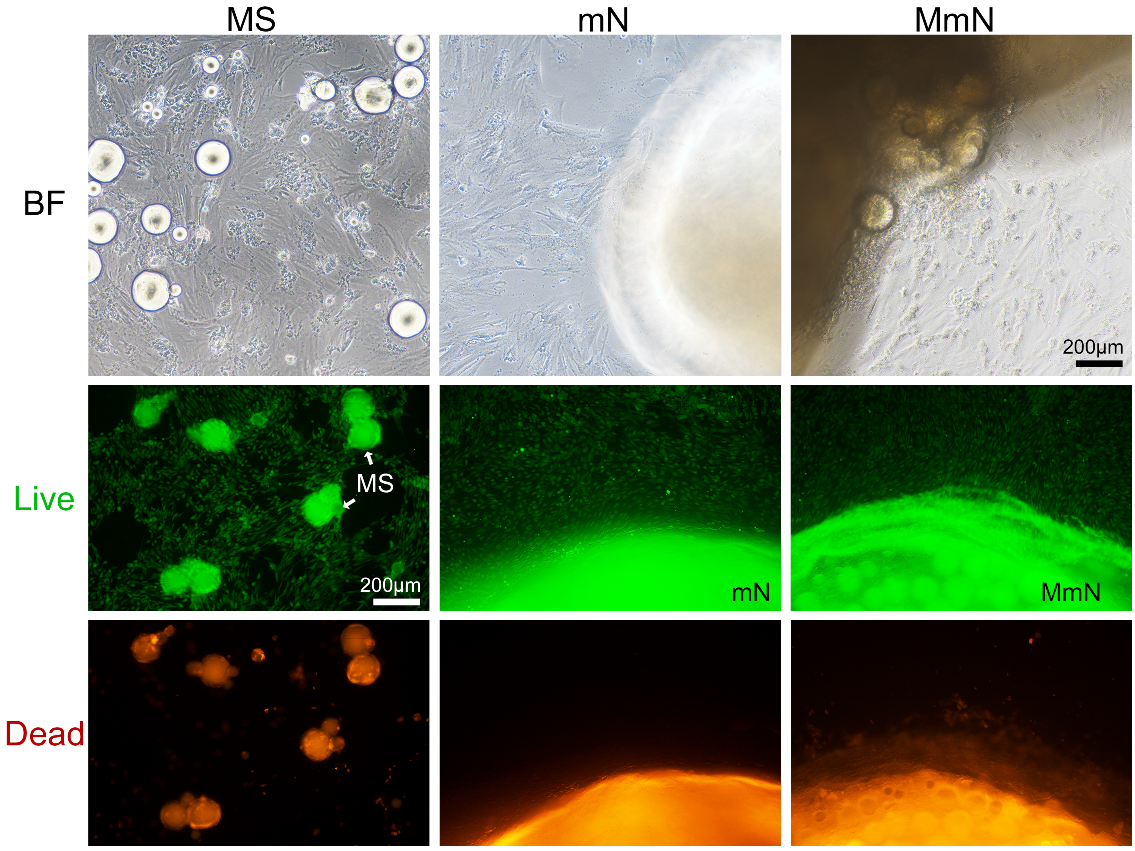


**Supplementary Figure 6.** Cell Morphologies and the live & dead staining of rBMSCs after co-culturing with different materials for 3 days. BF: Bright field.

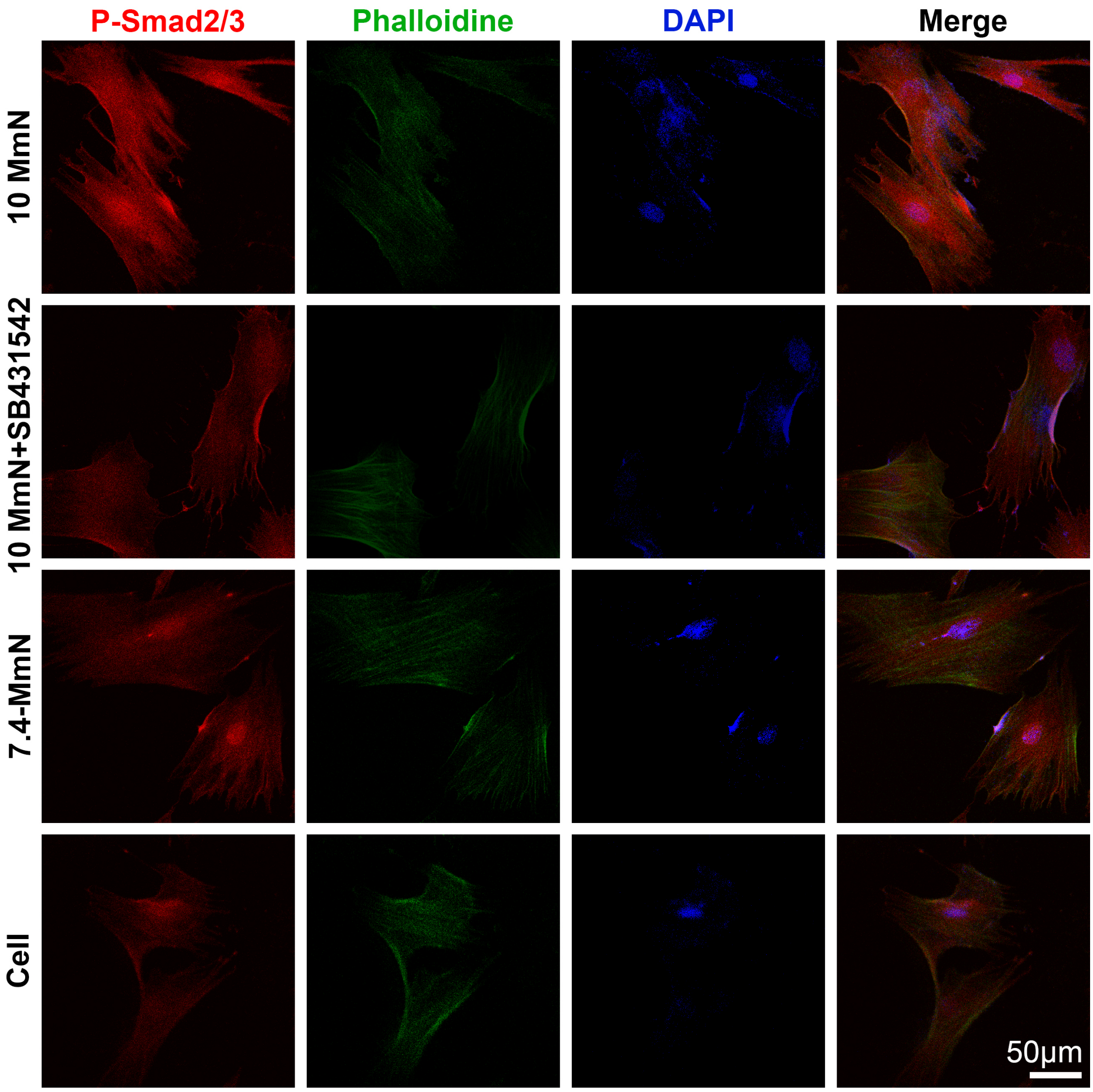


**Supplementary Figure 7.** The addition of 10% treated rat serum for the smad pathway activation by confocal microscopy.

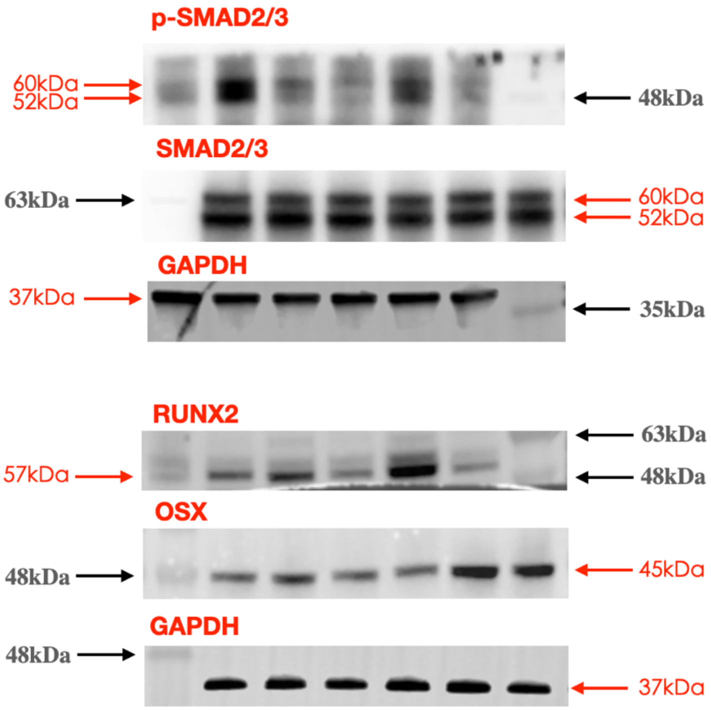


**Supplementary Figure 8.** Original images of western blotting.

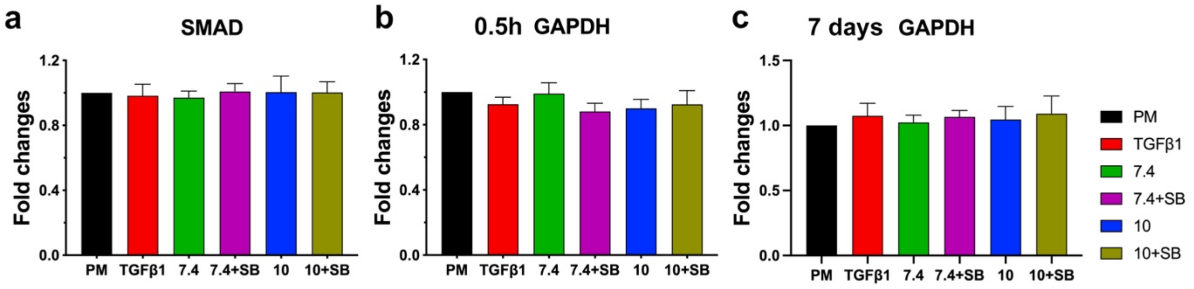


**Supplementary Figure 9.** Quantified results of western blotting. Data are presented as mean+/- standard deviation.

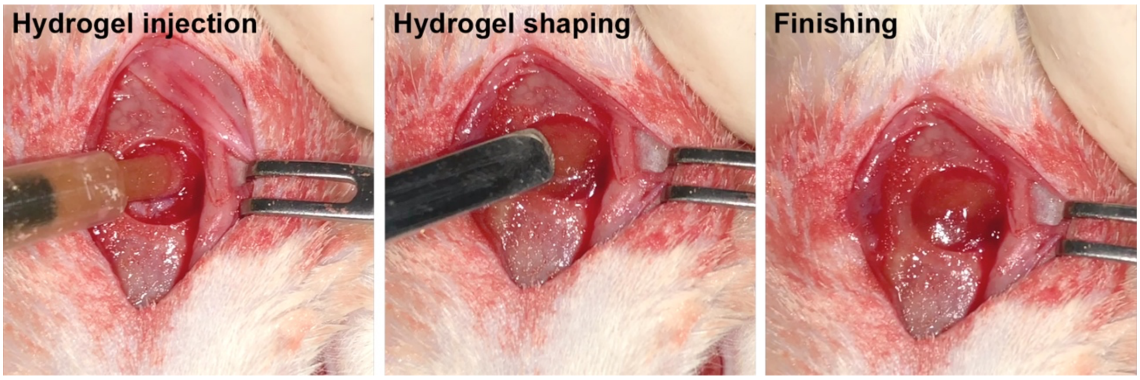


**Supplementary Figure 10.** Digital images showing the processes of hydrogel filling. The movie showing hydrogel infusion and shaping is shown in the Supplementary Video 2.

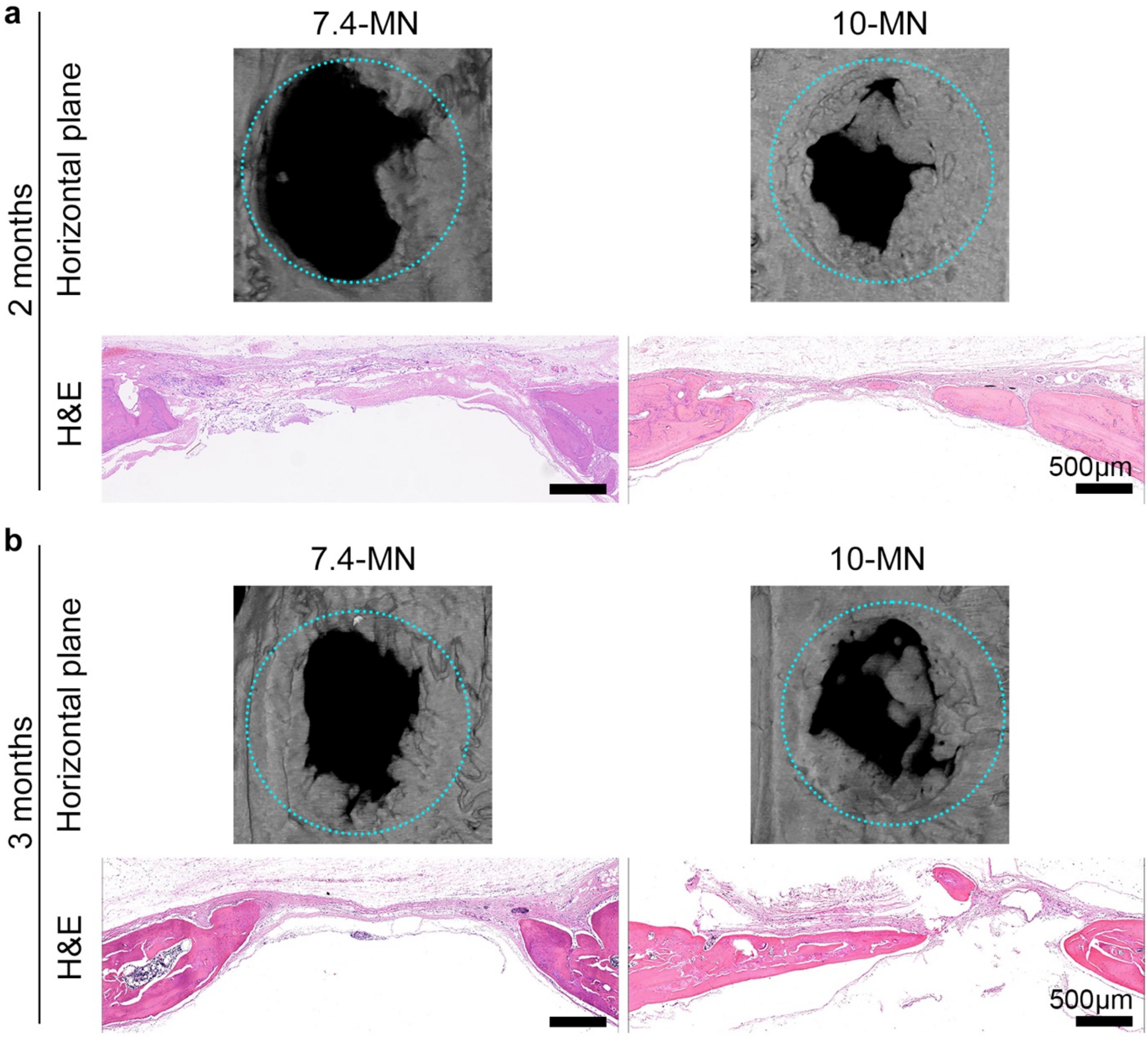


**Supplementary Figure 11.** Representative μCT-reconstructed 3D images in the horizontal plane and H&E staining of decalcified bone sections in the coronal plane of control groups, in a rat calvarial critical-sized defects of 5 mm, after 2 and 3-month treatments.

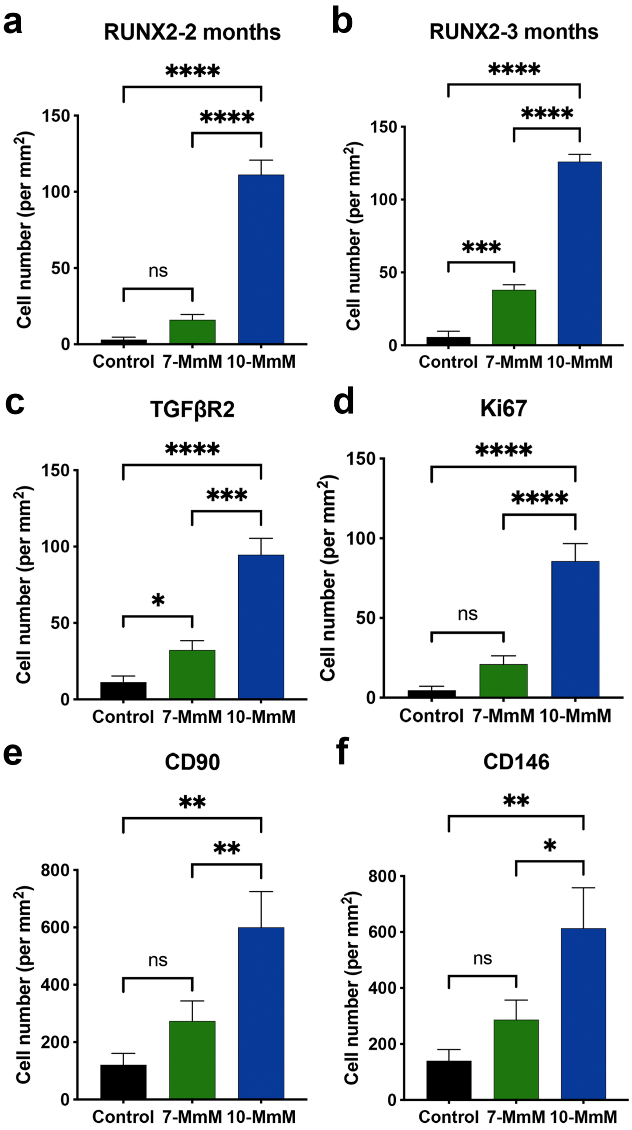


**Supplementary Figure 12.** Quantified results of immunohistochemical results. Data are presented as mean+/- standard deviation.

**Supplementary Tables**

Supplementary Table 1. pH Changes of rat serum after hydrogel treatments.

| Time(h) \ Sample | Control | 7.4-MN | 7.4-MmN | 10-MN | 10-MmN |
|---|---|---|---|---|---|
| 0.5 | 7.40 ± 0.02 | 8.51 ± 0.04 | 8.56 ± 0.02 | 9.81 ± 0.03 | 9.84 ± 0.01 |
| 10 | | 8.49 ± 0.07 | 8.47 ± 0.06 | 9.90 ± 0.04 | 9.87 ± 0.02 |
| 24 | | 8.38 ± 0.12 | 8.54 ± 0.07 | 9.80 ± 0.05 | 9.75 ± 0.01 |
| 48 | | 8.38 ± 0.11 | 8.54 ± 0.05 | 9.80 ± 0.02 | 9.75 ± 0.05 |

*n* = 3 independent experiments. The mean ± SD represents the data.

Supplementary Table 2. Primary and secondary antibodies.

| **Products** | **Product ID** | **Suppliers** |
|---|---|---|
| Anti-Phospho-Smad2/Smad3 Rabbit mAb | 8828 | CST |
| Anti-Smad2/3 Rabbit mAb | 8685 | CST |
| Anti-GAPDH Rabbit mAb | 2118 | CST |
| Anti-RUNX2 Rabbit monoclonal | ab236639 | Abcam |
| Anti-Sp7/Osterix Rabbit monoclonal | ab209484 | Abcam |
| Goat Anti-Rabbit IgG | ab6721 | Abcam |
| Anti-TGFβR2 Mouse mAb | 66636-1-lg | Proteintech |
| Anti-Ki67 Rabbit mAb | ab16667 | Abcam |
| Anti-CD90 Mouse mAb | ab181469 | Abcam |
| Anti-CD146 Rabbit mAb | ab75769 | Abcam |

Supplementary Table 3. Primers sequences of rat genes

| Genes | Forward sequences | Reverse sequences |
|---|---|---|
| *Gapdh* | 5’-CGGACAGGATTGACAGATTGATAGC-3’ | 5’-TGCCAGAGTCTCGTTCGTTATCG-3’ |
| *Osx* | 5’-ATGGCGTCCTCTCTGCTTG-3’ | 5’-TGAAAGGTCAGCGTATGGCTT-3’ |
| *Runx2* | 5’-AGCGGACGAGGCAAGAGTTT-3’ | 5’-CTGTCTGTGCCTTCTTGGTTCC-3’ |
| *Alp* | 5’-GGCTCTGCCGTTGTTTCTCT-3’ | 5’-AAGGTGCTTTGGGAATCTGC-3’ |
| *Col-1* | 5’-TGCTCCTACAAAGCTGTCTCC-3’ | 5’-GATGGACTCAACGGTCTCCC-3’ |

**Supplementary Videos**

Supplementary Video 1 & 2 to this article can be found online.
