# Supplementary material for: Alkaline shear-thinning micro-nanocomposite hydrogels initiate endogenous TGFβ signaling for in situ bone regeneration
Source: NPJ Regen Med. 2023 Oct 13;8:56. doi: 10.1038/s41536-023-00333-z (PMC10575889; doi:10.1038/s41536-023-00333-z)
Supplement: Supplementary file 2 — Reporting Summary [file 41536_2023_333_MOESM2_ESM.pdf]

nature portfolio

Corresponding author(s): Prof. Yongsheng Zhou

Last updated by author(s): Sep 23, 2023

Reporting Summary

Nature Portfolio wishes to improve the reproducibility of the work that we publish. This form provides structure for consistency and transparency in reporting. For further information on Nature Portfolio policies, see our Editorial Policies and the Editorial Policy Checklist.

Statistics

For all statistical analyses, confirm that the following items are present in the figure legend, table legend, main text, or Methods section.

n/a	Confirmed	
☐	☒	The exact sample size (*n*) for each experimental group/condition, given as a discrete number and unit of measurement
☐	☒	A statement on whether measurements were taken from distinct samples or whether the same sample was measured repeatedly
☐	☒	The statistical test(s) used AND whether they are one- or two-sided<br>*Only common tests should be described solely by name; describe more complex techniques in the Methods section.*
☐	☒	A description of all covariates tested
☐	☒	A description of any assumptions or corrections, such as tests of normality and adjustment for multiple comparisons
☐	☒	A full description of the statistical parameters including central tendency (e.g. means) or other basic estimates (e.g. regression coefficient) AND variation (e.g. standard deviation) or associated estimates of uncertainty (e.g. confidence intervals)
☐	☒	For null hypothesis testing, the test statistic (e.g. *F*, *t*, *r*) with confidence intervals, effect sizes, degrees of freedom and *P* value noted<br>*Give P values as exact values whenever suitable.*
☒	☐	For Bayesian analysis, information on the choice of priors and Markov chain Monte Carlo settings
☒	☐	For hierarchical and complex designs, identification of the appropriate level for tests and full reporting of outcomes
☒	☐	Estimates of effect sizes (e.g. Cohen's *d*, Pearson's *r*), indicating how they were calculated

Our web collection on statistics for biologists contains articles on many of the points above.

Software and code

Policy information about availability of computer code

Data collection	Skyscan for µCT scanning ; ZEN (Zeiss) for confocal image collection; Gen5 for absorbance; Kinexus Pro+ rheometer for mechanical tests; SU8010 for SEM (HITACHI) and JEOL 1400 for TEM;
Data analysis	µCT analysis using NRecon, CTVox and CTAn softwares; confocal image editing using ZEN (Zeiss); data visualization using Graph Pad Prism 9, Photoshop 2023); Scheme drawing using Adobe Dimension and Photoshop 2023;

For manuscripts utilizing custom algorithms or software that are central to the research but not yet described in published literature, software must be made available to editors and reviewers. We strongly encourage code deposition in a community repository (e.g. GitHub). See the Nature Portfolio guidelines for submitting code & software for further information.

Data

Policy information about availability of data

All manuscripts must include a data availability statement. This statement should provide the following information, where applicable:

- Accession codes, unique identifiers, or web links for publicly available datasets
- A description of any restrictions on data availability
- For clinical datasets or third party data, please ensure that the statement adheres to our policy

All data needed to evaluate or reproduce the conclusions in the paper are present in the paper and/or the Supplementary Information. Any other requests for raw

or processed data will be reviewed by Peking University National Engineering Laboratory for Digital and Material Technology of Stomatology to verify whether the data requested are subject to any intellectual property or confidentiality obligations.

Research involving human participants, their data, or biological material

Policy information about studies with human participants or human data. See also policy information about sex, gender (identity/presentation), and sexual orientation and race, ethnicity and racism.

Reporting on sex and gender	No human participants were used.
Reporting on race, ethnicity, or other socially relevant groupings	None
Population characteristics	None
Recruitment	None
Ethics oversight	None

Note that full information on the approval of the study protocol must also be provided in the manuscript.

Field-specific reporting

Please select the one below that is the best fit for your research. If you are not sure, read the appropriate sections before making your selection.

☒ Life sciences ☐ Behavioural & social sciences ☐ Ecological, evolutionary & environmental sciences

For a reference copy of the document with all sections, see nature.com/documents/nr-reporting-summary-flat.pdf

Life sciences study design

All studies must disclose on these points even when the disclosure is negative.

Sample size	No statistical method was used to predetermine a sample size. Sample size was determined according to previously published papers in related field and prior experience in our laboratory. Mice sample size was estimated by pilot experiments that presented trends of effects and their size. In most cases, an n=3 was the minimal amount used. Specific details were available in Methods section.
Data exclusions	The data was excluded when mice were dead or suffered from serious post-srugery weight loss.
Replication	At least 3 biological independent samples were performed for each experiment. All replications were successful.
Randomization	Samples were randomly assigned to experimental or control groups.
Blinding	All the surgical treatments and the functional experiments were done by blinded investigators.

Reporting for specific materials, systems and methods

We require information from authors about some types of materials, experimental systems and methods used in many studies. Here, indicate whether each material, system or method listed is relevant to your study. If you are not sure if a list item applies to your research, read the appropriate section before selecting a response.

Materials & experimental systems

n/a	Involved in the study	
☐	☒	Antibodies
☐	☒	Eukaryotic cell lines
☒	☐	Palaeontology and archaeology
☐	☒	Animals and other organisms
☒	☐	Clinical data
☒	☐	Dual use research of concern
☒	☐	Plants

Methods

n/a	Involved in the study	
☒	☐	ChIP-seq
☒	☐	Flow cytometry
☒	☐	MRI-based neuroimaging

Antibodies

Antibodies used	All antibodies are listed and in detail described in Supplementary Table 2.
Validation	All primary and secondary antibodies were validated on perspective manufacturers' websites with cited prior publications using (IHC,IF, WB)and species.

Eukaryotic cell lines

Policy information about cell lines and Sex and Gender in Research

Cell line source(s)	Rat BMSCs were purchased from Cyagen (OriCell® RASMX-01001).
Authentication	The cells have been authenticated by the supplier and they were used as received.
Mycoplasma contamination	The cells were not tested for mycoplasma contamination.
Commonly misidentified lines (See ICLAC register)	None

Animals and other research organisms

Policy information about studies involving animals; ARRIVE guidelines recommended for reporting animal research, and Sex and Gender in Research

Laboratory animals	6-to-8-week-old male Sprague Dawley (SD) rats
Wild animals	No wild animals were used.
Reporting on sex	Male
Field-collected samples	No samples were collected from the field.
Ethics oversight	The protocols were approved by the ethics committee of Peking University Health Science Center (Approval number: LA2019019). All the authors comply with all relevant ethical regulations.

Note that full information on the approval of the study protocol must also be provided in the manuscript.
